# Information Content of Prefrontal Cortex Activity Quantifies the Difficulty of Narrated Stories

**DOI:** 10.1038/s41598-019-54280-1

**Published:** 2019-11-29

**Authors:** Soheil Keshmiri, Hidenobu Sumioka, Ryuji Yamazaki, Masahiro Shiomi, Hiroshi Ishiguro

**Affiliations:** 1 Advanced Telecommunications Research Institute International (ATR), Kyoto, Japan; 2 Symbiotic Intelligent Systems Research Center, Institute for Open and Transdisciplinary Research Initiatives, Osaka University, Osaka, Japan; 3 Graduate School of Engineering Science, Osaka University, Osaka, Japan

**Keywords:** Social behaviour, Human behaviour

## Abstract

The ability to realize the individuals’ impressions during the verbal communication allows social robots to significantly facilitate their social interactions in such areas as child education and elderly care. However, such impressions are highly subjective and internalized and therefore cannot be easily comprehended through behavioural observations. Although brain-machine interface suggests the utility of the brain information in human-robot interaction, previous studies did not consider its potential for estimating the internal impressions during verbal communication. In this article, we introduce a novel approach to estimation of the individuals’ perceived difficulty of stories using the quantified information content of their prefrontal cortex activity. We demonstrate the robustness of our approach by showing its comparable performance in face-to-face, humanoid, speaker, and video-chat settings. Our results contribute to the field of socially assistive robotics by taking a step toward enabling robots determine their human companions’ perceived difficulty of conversations, thereby enabling these media to sustain their communication with humans by adapting to individuals’ pace and interest in response to conversational nuances and complexity.

## Introduction

Gone are the days when robots were sitting on the factory floors to perform tasks whose instructions were hardcoded in a great detail flawlessly. As the field of robotics matures, human society witnesses a growing integration of these media in individuals’ daily lives and activities. In fact, today’s robotics is less about assisting humans perform their physical tasks and more about facilitating their social interaction^1^. This observation is evident in growing adaptation of these media in such broad social domains as early child education^2,3^ and elderly care^4,5^. Pivotal to these applications is the ability for these agents to engage in social interaction^6^ and therefore solutions to such hard problems as learning the social norms and dynamics form the foundation for enabling robots understand the intentions of their human companion, thereby allowing them to achieve a sustainable long-term interaction.

However, obtaining these abilities by only observing the human behaviour is not sufficient considering the fact that behavioural cues can often be interpreted in different ways which is even more so during a verbal communication. For example, a frowning face during a conversation can be construed as sign of attentiveness or it may signal the person’s difficulty in following its content. Interestingly, deciphering such cues becomes even harder once we take into account the ability of individuals to disguise their emotions (e.g., smiling in a stressful situation). In the same vein, the fact that such reactions are highly subjective (i.e., they differ from person to person) makes facial analysis approaches^7^ fall short in decoding the cues associated with verbal communication: shy students that find a lecture difficult can shun the clarifying questions by acting as if they were following the lecture.

On the other hand, the human brain as the source of these subjective and internalized states can provide more reliable information about them. The brain activity cannot be easily suppressed or manipulated and therefore the information that is reflected in such activities has the potential to quantify individuals’ feelings. However, despite substantial progress in utilization of the brain information in such applications as brain machine interface (BMI)^8^ and human-robot interaction (HRI)^9,10^, there appears to be a paucity of research (to the best of our knowledge) on the use of brain information for estimating individuals’ assessment of the verbal communication (e.g., its difficulty) during HRI. Robots can particularly benefit from such an ability while interacting with overstressed persons and individuals with selective mutism disorders.

In this article, we propose to estimate the individuals’ perceived difficulty of a verbal communication by quantifying the information content of their prefrontal cortex (PFC) activity. We use the term “perceived difficulty” to refer to the cognitive load that a person’s PFC may endure during such tasks as language processing^11^, social cognition^12^, and story comprehension^13–15^. In this respect, functional imaging techniques in conjunction with such tasks as mental arithmetic (MA)^16^ and n-back^17^ have enabled researchers to shed light on PFC functioning and change in its activity in response to varying cognitive load^16–18^. Considering these findings, we expect that these tasks can also be useful in estimating the perceived difficulty of a more cognitively demanding task like verbal communication.

We also introduce a novel information-theoretic approach for quantification of such cognitive loads. The choice of information is motivated by the following three observations. First, information is an unbiased measure of association between interacting processes^19^ (e.g., change in the brain activity in response to varying task’s difficulty) and hence an attractive choice for brain mapping^20^ as well as the modeling of its inherent complexity^21–24^. Second, information allows for a more robust handling of such confounders as residual brain activity prior to the start of the task period (also known as resting state^25^). This ability plays a central role in preventing an overestimation of the cognitive load due to the prior brain activity that is not induced by the task. Third, brain activation can take place in a shorter time span (i.e., faster) in case of easier than more difficult tasks^26^ and therefore its differential activities can simply be averaged out^27–29^ if the incurred variability is not accounted for^30–32^. This hinders the ability to differentiate the activation patterns that are induced by tasks with varying level of difficulty. On the other hand, information of a continuous random variable is a function of the variance than the mean^33^, p. 182] and therefore can preserve the variability of such time series as brain activity^34^. This, in turn, makes information well-suited for scenarios in which higher variability in individuals’ brain responses is expected (e.g., verbal communication). Therefore, we expect that an information-based quantification of the individuals’ PFC activation to form a reliable biomarker for measuring the cognitive load that is associated with the difficulty of a verbal communication.

In our approach, we first determine a decision boundary that distinguishes between the incurred cognitive loads by one- and two-back auditory tasks on individuals’ PFC (acquired by near-infrared spectroscopy (NIRS)). In this task, the participants are instructed to respond (e.g., through mouse clicks) to the repeated patterns in numerical sequences (e.g., sequential i.e., n = 1 or one-back and every-other repetition i.e., n = 2 or two-back) that are presented auditorily. We use n-back in our study due to its demonstrated ability in inducing differential cognitive load on PFC^35^ as well as its utility in quantifying the PFC activity in response to individuals’ change in mood^18,36^. Then in a realtime storytelling scenario, we use this boundary to estimate the individuals’ perceived difficulty of narrated stories, thereby interpreting their perceived difficulty of stories based on induced cognitive load by n-back auditory task.

Our contributions are threefold. First, we introduce a novel information-theoretic approach to quantification of the induced cognitive load on PFC. We present the effectiveness of our approach in quantification of the PFC activity during a WM task. Our results show a substantial improvement on the previous findings^17,37^. Second, We demonstrate the utility of our approach in estimation of the individuals’ perceived difficulty of the verbally communicated content in a humanoid-mediated storytelling scenario. Third, we provide evidence for robustness of our approach through comparative analysis of its performance in face-to-face, humanoid, speaker, and video-chat system media settings.

In our view, the use of brain information can advance the HRI research on modeling of the human behaviour by providing invaluable information about mechanisms that underlie human behavioural responses. For instance, brain activity can be used as neurophysiological feedbacks about individuals’ mental states^6^ in multimodal modeling of human behaviour^38^. This, in turn, can open a new venue for formal analysis of a robotic ToM^39^ that (in addition to behavioural observations) builds upon critical implications of the humans’ neurological responses during interaction with their synthetic companions.

## Methods

Our approach comprises of three steps: (A) information-theoretic formulation of the cognitive load (*CL*), (B) determination of a decision boundary that identifies the *CL* quantities that are uniquely associated with differing WM task’s level, (C) realtime estimation of the perceived difficulty of the verbally communicated content. In what follows, we explain each step in details.

### Information-theoretic formulation of cognitive load

This step consists of two components: the “quantification of the cognitive load” that estimates the induced cognitive load on the PFC in response to external stimuli per estimation step and the “constrained updating of the induced cognitive load” that dictates an update rule to reduce the effect of the PFC activity’s fluctuations on such a quantification.

#### Quantification of the cognitive load

Let *X*_*τ*_ represent the time series associated with the task period’s PFC activity at estimation step *τ*. Let *B* be the baseline (i.e., resting state) time series that represent the frontal brain activity prior to the start of the task. Furthermore, let *H*(*X*_*τ*_) represent the entropy of *X*_*τ*_ (i.e., its average information content). Although *H*(*X*_*τ*_) quantifies the PFC’s cognitive load (*CL*)^40^ at *τ*, it is an overestimation of *CL* if PFC’s residual activity that is carried over from the resting period is not attenuated. It is also crucial to observe that such a residual effect cannot be attenuated by mere subtraction of the expected resting state’s activity (i.e., *μ*_*B*_) from *X*_*τ*_ due to the invariant of information to translation [^41^, Theorem 8.6.3, p. 253]. Therefore, we quantify the PFC’s cognitive load at estimation step *τ* i.e., *CL*(*X*_*τ*_) through conditioning of the PFC activity at *τ* with respect to its activity prior to the start of the task:

1 $$CL({X}_{\tau })=H({X}_{\tau }|B)=H({X}_{\tau })-MI({X}_{\tau };B)$$

where MI(*X*_*τ*_; *B*) represents the mutual information between PFC activity during the task period at estimation step *τ* and its activation pattern during the resting period.

#### Constrained updating of the induced cognitive load

Neuroscientific findings imply that the brain activity occurs in sparse transient^42^. In other words, observed brain activities are subject to fluctuation. Considering the direct correspondence between information and the variation^33,40^, such a sparsity can directly affect the calculated *CL* as formulated in Eq. (1) which, in turn, can result in a false belief about the overall task-induced cognitive load on PFC due to the accumulation of such over/underestimations of *CL*. In other words, fluctuating patterns in PFC activity can result in rapid changes in signal variability whose discrimination from desirable task-induced changes in PFC activation might not be trivial if one only rely on the computed *CL*. For instance, an increase/decrease in *CL* at a given time might solely be explained by a short-lived fluctuation and not the effect of the task per se on PFC. In such a scenario, simply following the computed *CL* can lead to a false conclusion since a small number of such rapid and short-lived incremental/decremental fluctuations can cancel out and average the actual effect of the task on PFC activity.

Above observations identify the need for additional measures to validate the correspondence between potential differences between two consecutive *CL*s. More importantly, these measures must take into account the pattern of PFC activity associated with these consecutive *CL*s to verify whether their observed differences are in fact due to a substantial variation than a mere short-lived fluctuation. In other words, they must allow for constraining the observed differences between two consecutive *CL*s with the level of change in their respective PFC variability.

Interestingly, these fluctuations can conveniently be accounted for through realization of the MI: a measure of the shared information among interacting processes [^41^, p. 19 and p. 251]. Specifically, rewriting Eq. (1) as *MI*(*X*_*τ*_; *B*) = *H*(*X*_*τ*_)−*CL*(*X*_*τ*_) it becomes apparent that an increase/decrease in the cognitive load must, in principle, be accompanied with its corresponding decrease/increase in mutual information between *X*_*τ*_ and *B*. In fact, if the interacting processes belonged to a well-defined parametric distribution it would have sufficed to solely check for *MI*(*X*_*τ*−1_; *B*) and *MI*(*X*_*τ*_; *B*) to discriminate between potential fluctuations and the legitimate variations in the task-induced cognitive load. However, extent of the brain dynamics that borders with chaotic system^43^ in conjunction with varying complexity of naturalistic tasks (e.g., conversational nuances and change in difficulty of their contents) do not warrant the utility of such simplifying assumption as imposing a known parametric distribution on observed PFC activity during naturalistic scenarios.

Alternatively, we can verify whether above necessary condition in case of *MI* is also satisfied at the distribution level of these interacting processes, thereby bypassing any unwarranted assumption on their distributions. This can be achieved by utilization of the Kullback-Leibler divergence (*D*_*KL*_) that reflects the distance between the distribution of interacting processes [^41^, p. 19 and p. 251]. The utility of *D*_*KL*_ is realized by observing that any increase/decrease in MI due to a reduced/increased *CL* in *MI*(*X*_*τ*_; *B*) = *H*(*X*_*τ*_)−*CL*(*X*_*τ*_) indeed identifies an increase/decrease in resemblance between their distributions and therefore their reduced/increased divergence.

Therefore, we control for potential fluctuating patterns in PFC during the task performance by evaluating the difference between *CL*(*X*_*τ*−1_) and *CL*(*X*_*τ*_) through quantification of their respective *MI* and *D*_*KL*_ with respect to *B*, thereby constraining the updates of computed PFC’s cognitive load. Concretely, we directly use the result from Eq. (1) if the difference between *CL*(*X*_*τ*−1_) and *CL*(*X*_*τ*_) is warranted by their *MI* and *D*_*KL*_ with respect to *B* or, alternatively, we compensate for the potential fluctuation by averaging *CL*(*X*_*τ*−1_) and *CL*(*X*_*τ*_), weighted by their variation of information (VI)^44^:

2 $$CL({X}_{\tau })=\{\begin{array}{c}H({X}_{\tau }|B)\,\,\,\,(MI({X}_{\tau };B)\le MI({X}_{\tau -1};B)\,\,and\,\,{D}_{KL}({X}_{\tau };B) > {D}_{KL}({X}_{\tau -1};B))\\ \,\,\,\,\,\,or\,(MI({X}_{\tau };B) > MI({X}_{\tau -1};B)\,and\,{D}_{KL}({X}_{\tau };B)\le {D}_{KL}({X}_{\tau -1};B))\\ \alpha H({X}_{\tau -1}|B)+\beta H({X}_{\tau }|B)\,(otherwise)\end{array}$$

and:

3 $$\alpha =\frac{H({X}_{\tau -1}|{X}_{\tau })}{VI}$$

4 $$\beta =\frac{H({X}_{\tau }|{X}_{\tau -1})}{VI}$$

5 $$VI=H({X}_{\tau -1})+H({X}_{\tau })-2MI({X}_{\tau -1};{X}_{\tau })$$

6 $$=H({X}_{\tau -1}|{X}_{\tau })+H({X}_{\tau }|{X}_{\tau -1})$$

### Decision boundary determination

Let *S*1 and *S*2 denote the *CL*s that correspond to cognitive loads induced by two-level WM tasks. Computing the decision boundary 𝔻 between *S*1 and *S*2 is analogous to determining the midpoint between the *CL* quantities that uniquely fall within the *S*1 or *S*2 intervals i.e., the elements that are not members of their overlapping subset. Algorithm 1 outlines this process. It first sorts *S*1 and *S*2 in their descending and ascending orders (steps 1 and 2). Next, it finds the smallest *CL* in S1 that lies within the S2 interval (step 3) and the largest *CL* in *S*2 that is within *S*1 interval (step 4), thereby marking their overlapping partition. Then, it determines the immediate largest *CL* in *S*1 and the immediate smallest *CL* in *S*2 that are smaller and larger than *S*1’s and *S*2’s respective *CL*s that mark this overlapping partition. Last, it returns the average of these immediate largest and immediate smallest *CL*s as the decision boundary 𝔻 that separates the *CL*s associated with the disjoint *S*1 and *S*2 sets.

In this article, we utilize n-back auditory task as the WM task. In this case, *S*1 and *S*2 correspond to cognitive loads induced by one- and two-back WM tasks, respectively.

### Online estimation of the perceived difficulty of conversation

We utilize the calculated decision boundary 𝔻 for online estimation of the individuals’ perceived difficulty of the verbally communicated content. At every estimation step, our model calculates the *CL* of the current PFC activity time series. At the end of the verbal communication, it computes the median of these computed *CL*s and determines whether it is above or below the computed decision boundary 𝔻. Subsequently, it marks the individual’s perceived difficulty of the verbally communicated content as “difficult/easy” if this median is above/below 𝔻.

### Ethics statement

This study was carried out in accordance with the recommendations of the ethical committee of the Advanced Telecommunications Research Institute International (ATR) with written informed consent from all subjects in accordance with the Declaration of Helsinki. The protocol was approved by the ATR ethical committee (approval code:16-601-1).

## Experiments

We conducted two experiments to evaluate the utility of our model. In the first experiment, we verified whether our proposed measure of cognitive load can distinguish the PFC activation in response to low vs. high cognitive loads in one- and two- back WM task. This allowed us to evaluate the ability of our approach for differential quantification of the induced PFC activation in response to these tasks. It also allowed us to determine the decision boundary 𝔻 between differential cognitive loads on the PFC activity which we used in the second experiment.

The second experiment was for verification of the performance of our approach on estimation of the individuals’ perceived difficulty of the verbal communication in a naturalistic setting. For this purpose, we used storytelling as a first step toward decoding of the conversational communication since stories’ scripts can be kept intact and repeated to different individuals without any change in their contents, thereby allowing for the control of such confounders as subtle differences in conveyed information.

**Algorithm 1:**
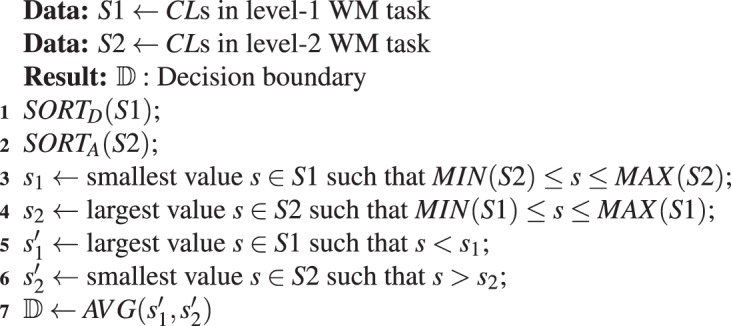
Determination of the decision boundary 𝔻. *SORT*_*D*_(.) and *SORT*_*A*_(.) sort the elements of their arguments in a descending and an ascending order, respectively. *AVG*(.) computes the average of its two arguments.

The “perceived difficulty” within the context of the first experimental paradigm then refers to the cognitive psychology notion of cognitive load: measurable change in WM capacity in processing information that is associated with controlled tasks that are specifically designed for WM excitation^17,40^. In the context of second experiment, on the other hand, it reflects the change in WM capacity at more subjective level (e.g., increase in WM information processing with respect to the change in stories’ difficulty) that is quantified by such fine-grained and well-designed class of WM tasks as n-back.

### Experiment 1: Discrimination of differential cognitive load in N-Back WM Task

#### Purpose

In this experiment, we validated the performance of our approach on quantification of the effect of the WM tasks on PFC activity. Among such tasks, we chose n-back WM task since it forms a better basis for quantification of the verbally communicated contents, considering its effect on PFC^35^ and its ability in identifying the change in PFC activation in response to individuals’ emotions and change in mood^18^.

#### Participants

Thirty three younger adults (fourteen males and nineteen females, M = 30.96 years, SD = 10.84) participated in this experiment. Data from one male and one female were not recorded properly and were discarded. All participants were free of neurological and psychiatric disorders and had no history of hearing impairment. All experiments were carried out with written informed consents from all subjects. We used a job-offering site for university students to recruit our participants.

#### Paradigm

It included a seventy-second audio (in Japanese) sequences of numerical (1 through 9) one- and two-back WM tasks. Each session consisted of a one- and a two-back WM tasks. We kept the order as well as content of these WM tasks intact for all the participants. We used a speaker to play the audio sequences of numerical one- and two-back WM tasks to the participants. Every participant completed these two tasks. The participants responded to sequential (i.e., one-back) and every-other (in case of two-back) occurrences of these numerical values through mouse-clicks. We used PsychoPy for generating these audio one- and two-back WM tasks.

#### Procedure

Every participant first was seated in an armchair with proper head support in a sound-attenuated testing chamber and gave written informed consent in the experimental room. Then, a male experimenter explained the experiment’s full procedure to the participants. This included the total number of tasks in a session (i.e., a one-back followed by a two-back WM tasks), the duration of each task (i.e., seventy seconds per WM task), instructions on WM tasks procedure (i.e., periodic sequential (i.e., one-back) or every-other (i.e., two-back) occurrences of some of the numerical values), instructions on how to respond if the participants detected such reoccurrences (i.e., through the mouse-click at every detection), instructions about the one-minute rest period prior to the actual session (i.e., sitting still with eyes closed), and the content of the audio sequences (i.e., numerical values 1 through 9). The experimenter also asked the participants to stay focused on listening to the one- and two-back sequences that were played back to the participants through a computer speaker and then began the experimental session. Every one- and two-back WM task started by recording a one-minute rest data which was followed by its seventy seconds WM task period. We recorded the participants’ frontal brain activity time series throughout these tasks’ periods (including their respective one-minute resting).

**Figure 1:**
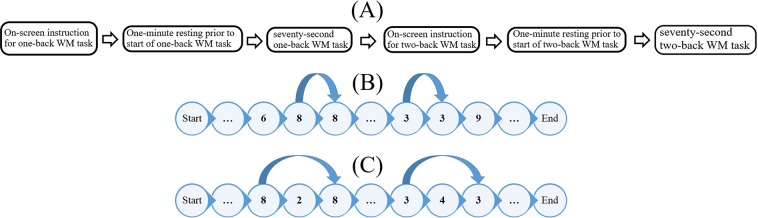
(**A**)Schematic of one- and two-back WM tasks session. (**B**) One-back WM task (**C**) Two-back WM task.

Once the participants were ready, the experimenter asked them to follow the instructions on the computer screen in front of them (Fig. 1(A)). The instructions on the display informed the participants that they participate in a one- and a two-back WM tasks, that each WM task was seventy seconds long, that the task period was proceeded with a one minute resting period during which they needed to close their eyes and relax as much as possible, and that once this resting period was over a voice (recorded voice of the experimenter through the computer speaker) would announce the start of the WM task after which the task would immediately begin. These instructions also provided the participants with an audio-visual example of the task that was about to begin. For instance, in the case of one-back it displayed a short sequence of numbers which were (in sequential fashion) highlighted by a square around the digit that was being read out and explaining how the value that was just read out was related to the value one-back before (Fig. 1(B)). The participants then started with the resting period of the one-back WM task and immediately engaged in this task once the end of the resting period and the start of the WM task was announced by the voice. For the one-back task, there were ten numerical values that were repeated in one-back fashion (Fig. 1(B)). Once one-back was over, the voice announced the end of the task and instructed the participants to follow the on-screen information for two-back task (similar to the one-back but this time the instruction of the task was about the two- than one-back WM task). The participants then started the two-back WM task which began by its one-minute resting period at the end of which the voice asked the participants to open their eyes and that the task was about to start. Two-back WM task also (similar to one-back) included the repetition of ten numerical values in a two-back fashion (Fig. 1(C)). Once the two-back WM task was over, the experimenter removed the NIRS device from the forehead of the participants and guided them out side the experimental room.

#### Data acquisition

**Figure 2:**
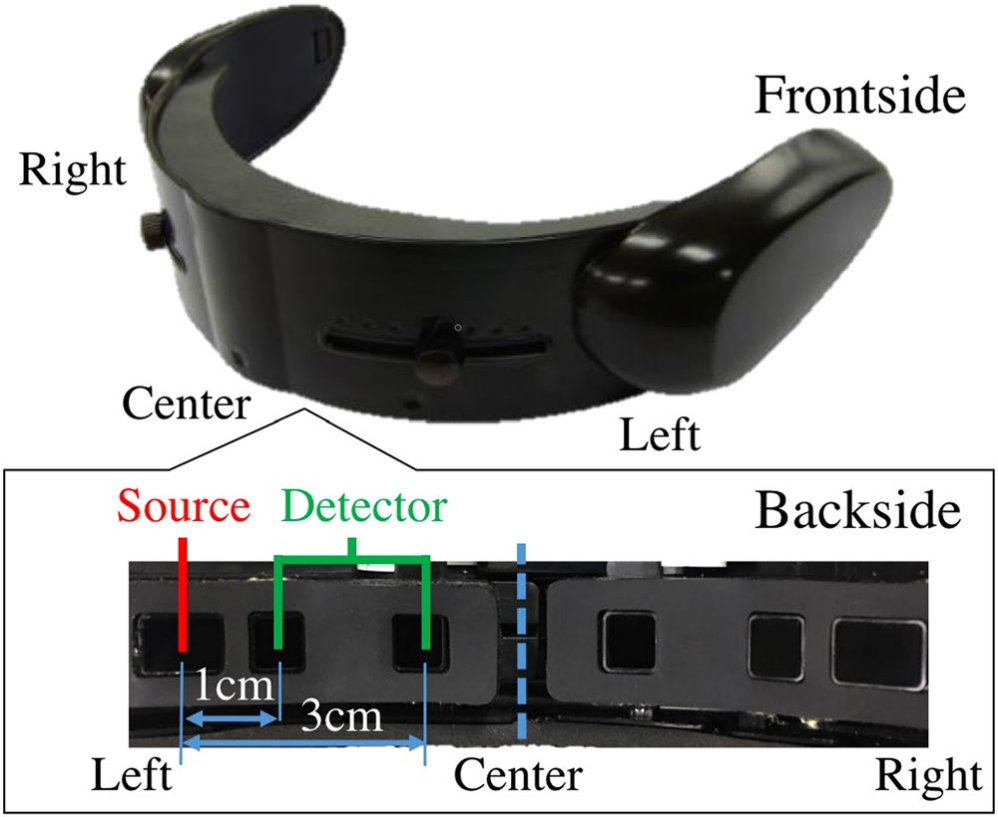
fNIRS device in present study. Bottom subplot on left shows arrangement of source-detector of four channels of this device. Distances between short (i.e., 1.0 cm) and long (i.e., 3.0 cm) source and detector of left and right channels are shown.

We used functional near infrared spectroscopy (fNIRS) to collect the frontal brain activity of the participants and acquired their NIRS time series data using a wearable optical topography system called “HOT-1000,” developed by Hitachi High-Technologies Corp. (Fig. 2). Participants wore this device on their forehead to record their frontal brain activity through detection of the total blood flow by emitting a wavelength laser light (810 nm) at a 10.0 Hz sampling rate. Data acquisition was carried out through four channels (*L*1, *L*3, *R*1, and *R*3, Fig. 2). Postfix numerical values that are assigned to these channels specify their respective source-detector distances. In other words, *L*1 and *R*1 have a 1.0 cm source-detector distance and *L*3 and *R*3 have a 3.0 cm source-detector distance. Note that whereas a short-detector distance of 1.0 cm is inadequate for the data acquisition of cortical brain activity (e.g., 0.5 cm^45^, 1.0 cm^46^, 1.5 cm^47^, and 2.0 cm^48^), 3.0 cm is suitable^45,47^. Therefore, we mainly report the result with the data from L3 in present study and the result with R3 is shown in Supplementary Materials (SM).

#### Data processing

We first attenuated the effect of systemic physiological artefacts^49^ (e.g., cardiac pulsations, respiration, etc.) using a one-degree polynomial Butterworth filter with 0.01 and 0.6 Hz for low and high bandpass which was then followed by performing the linear detrending on the data. We then attenuated the effect of the skin blood flow (SBF) using an eigen decomposition technique^50^. This approach considers the first three principal components of all NIRS recorded channels of the participants’ frontal brain activity during rest period to represent the SBF. Subsequently, it eliminates the SBF effect by removing these three components from participants’ NIRS time series in the task period. Although Sato *et al*.^47^ suggested that the use of first principal component than first three components appeared to be sufficient for SBF attenuation, Keshmiri *et al*.^51^ demonstrated that the use of first two principal components resulted in both significantly higher SBF attenuation as well as more cortical activity’s information preservation. Therefore, we followed^51^ and removed the first two principal components of the respective resting period of the participants from the NIRS time series of their frontal brain activity that was recorded during the task period. It is worth emphasizing that we used the same measurement settings (i.e., same equipment, number of measurement channel, and its position) as in Keshmiri *et al*.^51^ Similar to our NIRS recording, Zhang *et al*.^50^ also used 3.0 cm source-detector distance channels. Cooper *et al*.^52^ showed that this filter also attenuates the motion artefact (e.g., head motion).

While quantifying the PFC activation, we used twenty-second NIRS time series segments of participants’ PFC activation with ten-second of overlap between every two consecutive segments to calculate the *CL*s at every ten-second estimation step. We used our mathematical model in Section 2.1 for *CL* computation. For the first segment in the task period, we considered its overlap with the last ten seconds of the rest period. We used the last twenty-second of the rest period’s NIRS time series for each participant in Eq. (1). The ten-second estimation step resulted in seven *CL*s in case of one- and two-back WM tasks (per task).

#### Analysis

First, we computed the medians (per participant per task) of the *CL*s for the one- and the two-back WM tasks. Then, we applied Wilcoxon rank sum on these medians to determine the differential significance between these WM tasks’ *CL*s. Next, we used these medians to determine the discrimination accuracy of our model’s *CL*s in differentiating between one- and two-back WM tasks. We computed the accuracy of our model using Mdn_*two*−*back*_ &gt; Mdn_*one*−*back*_ (Mdn stands for median) criterion per participant. We also computed the Spearman correlation between these medians and the percentage of correct clicks by the participants. We scaled the participants’ number of clicks within [0, … 1] interval. Last, we computed the Spearman correlation between one- and two-back WM tasks’ medians. In order to determine the utility of our model, we also applied these analyses on participants’ average PFC activation (SM Section 1 for left-hemispheric and SM 2.1.2 for right-hemispheric PFC).

**Figure 3:**
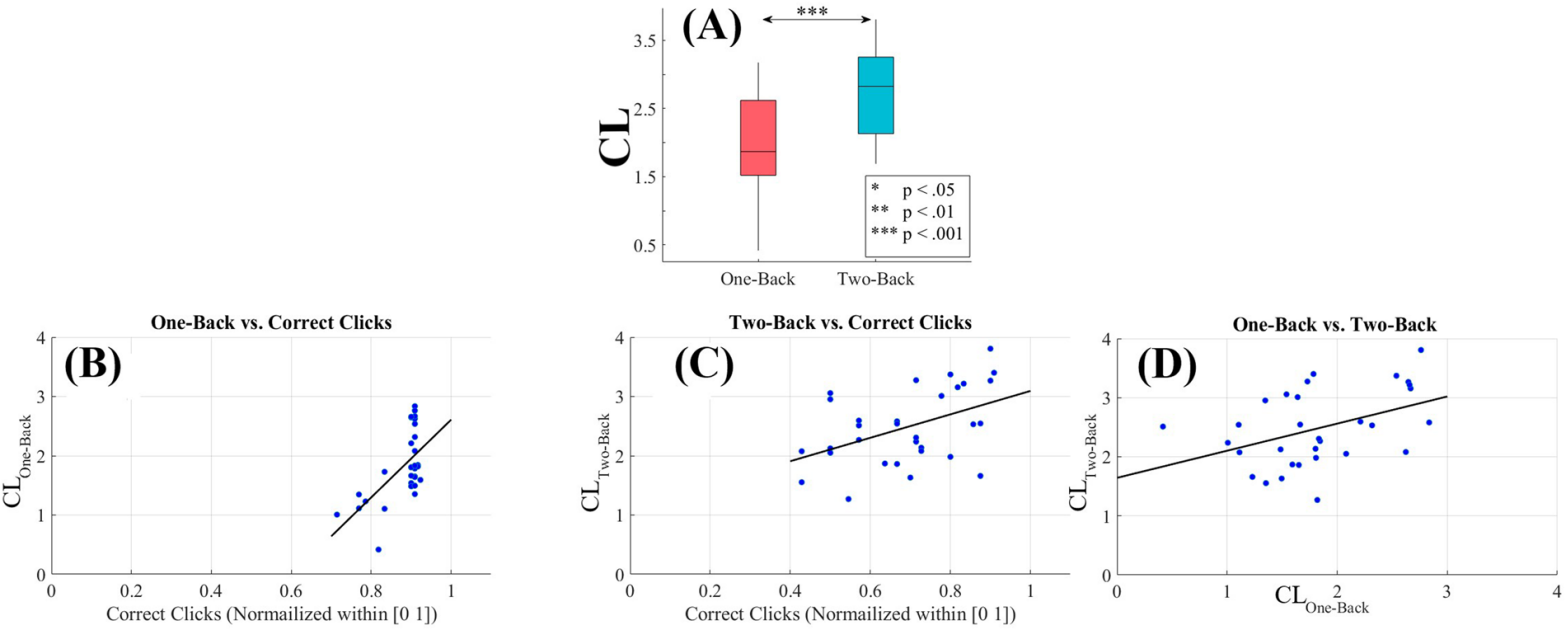
(**A**) Wilcoxon rank sum between participants’ CLs in one- and two-back WM tasks. Asterisks indicate the significant difference between these *CL*s. (**B**) Spearman correlation between participants’ *CL*s and the number of their correct clicks in one-back WM task. (**C**) Spearman correlation between participants’ *CL*s and the number of their correct clicks in response to two-back WM task. (**D**) Spearman correlation between participants’ *CL*s in one- and two-back WM tasks. In (**B**) and (**C**) correct clicks are normalized within [0, …, 1] interval. In these subplots, “CL” refers to the medians of the CLs for the two tasks.

To further examine whether the changes in participants’ CLs during two-back WM task were significantly associated with the cognitive load induced by this WM task period than being the residual effect from their one-back WM task period, we performed a one-sample bootstrap test of significance (10,000 simulation runs) at 99.0% confidence interval (CI) on the difference between participants’ CLs during two-back and one-back WM tasks (i.e., CL_*B*2_ − CL_*B*1_). We then considered the null hypothesis *H0: induced change in CLs by two-back WM task after the deduction of one-back’s CLs was non-significant* and tested it against the alternative hypothesis *H1: two-back WM task’s induced change in CLs after the deduction of one-back’s CLs was significant*. Since we considered CL_*B*2_ − CL_*B*1_, *H*0 and *H*1 then represented the situations in which CL_*B*2_ − CL_*B*1_ ≈ 0 (i.e., zero fell within the 99.0% confidence interval) and CL_*B*2_ − CL_*B*1_ > 0 (i.e., their 99.0% confidence interval was significantly above zero), respectively. It is worth noting that *H*1: CL_*B*2_ − CL_*B*1_ > 0 is equivalent to *H*1': CL_*B*1_ − CL_*B*2_ < 0. We reported the mean, standard deviation, and 99.0% confidence interval for left PFC in the main manuscript (for results associated with right PFC, see SM, Section 2 and Fig. 3).

Next, we computed the Spearman correlations between these CL_*B*2_ − CL_*B*1_ values and the participants’ correct clicks during the two-back WM task. We chose the participants’ correct clicks during two- than one-back WM tasks since CL_*B*2_ − CL_*B*1_ values reflected the quantitative changes in participants’ CLs associated with two-back WM task and after the reduction of participants’ CLs during one-back WM task period. We followed this by computing their 95.0% bootstrap (10,000 simulation runs) confidence intervals. For the bootstrap test, we considered the null hypothesis *H0: there was no correlation between CL*_*B2*_ − *CL*_*B1*_ *and participants’ correct clicks during two-back WM task* and tested it against the alternative hypothesis *H1: CL*_*B2*_ − *CL*_*B1*_ *significantly correlated with participants’ correct clicks during two-back WM task*. We reported the mean, standard deviation, and 95.0% confidence interval for this test. We also computed the p-value of this test as the fraction of the distribution that was more extreme than the actually observed correlation values. For this purpose, we performed a two-tailed test in which we used the absolute values so that both the positive and the negative correlations were accounted for.

Last, to ensure that the observed changes in the participants’ CLs during two-back WM tasks were due to the PFC activity during this WM task than artefacts (e.g., noise or an affine transformation of one-back’s CLs as a result of the underlying linear property of the hemodynamic responses^53,54^), we applied a one-sample bootstrap test of significance (10,000 simulation runs) at 99.0% confidence interval on the Kullback-Leibler divergence D_*KL*_ of participants’ CLs during two- and one-back WM tasks (i.e., D_*KL*_(CL_*B*2_, CL_*B*1_). we considered the null hypothesis *H0: difference in the distribution of CLs in two- and one-back WM tasks were non-significant* (hence one-back’s CLs can be used to explain the observed CLs during two-back WM task) and tested it against the alternative hypothesis *H1: distribution of CLs during two-back WM task was significantly different from one-back’s CLs*. We reported the mean, standard deviation, and 99.0% confidence interval for this test. It is worthy of note that whereas *H0* in this test was satisfied if zero fell within the computed D_*KL*_’s 99.0% confidence interval, *H1*’s satisfaction was associated with the case in which 99.0% confidence interval was significantly above zero (or equivalently significantly below zero in the case of D_*KL*_(CL_*B*1_, CL_*B*2_).

The earlier studies on n-back^35^ WM tasks and the language processing^11^ reported on a higher activation in left- than right-hemispheric PFC. On the other hand, the recent findings on the role of PFC in n-back^17^ and story comprehension^13–15^ indicate that such a distinction is not necessarily warranted. Therefore, we considered both left as well as right PFC in our study. However, we focused on the left PFC in the main manuscript since the activity in left PFC formed the common themes among these previous findings and provided the results pertinent to the right PFC in SM.

#### Results

Wilcoxon rank sum (Fig. 3(A)) identified a significant difference between the participants’ *CL*s in one- and two-back WM tasks (p < 0.001, W(60) = 3.59, r = 0.46, M_*one*−*back*_ = 1.83, SD_*one*−*back*_ = 0.59, M_*two*−*back*_ = 2.48, SD_*two*−*back*_ = 0.64). Our model achieved an 87.10% prediction accuracy for classification of these tasks. Table 1 summarizes these results.

**Table 1:** Wilcoxon rank sum along with the mean and standard deviation of the one- (*M*_1_ and *M*_2_) and two-back (*M*_2_ and *SD*_2_) *CL*s.

	p	W(60)	r	*M*_1_	*SD*_1_	*M*_2_	*SD*_2_	*Mdn*_2_ > *Mdn*_1_(%)
*CL*	<0.001	3.59	0.46	1.83	0.59	2.48	0.64	87.10

We calculated the accuracy of our approach in differentiating the participants’s PFC activation in response to one- and two-back WM tasks using the medians of one- and two-back *CL*s per participant.

We found a significant correlation between participants’ *CL*s and their number of correct clicks in response to one-back (Fig. 3(B)) WM task (r = 0.52, p < 0.01, M_*Clicks*_ = 0.88, SD_*Clicks*_ = 0.05). Similarly, this correlation was significant in two-back (Fig. 3(C)) WM task (r = 0.46, p < 0.01, M_*Clicks*_ = 0.69, SD_*Clicks*_ = 0.15). Last, we observed (Fig. 3(D)) a significant correlation between participants’ *CL*s in one- and two-back WM tasks (r = 0.41, p < 0.03).

**Figure 4:**
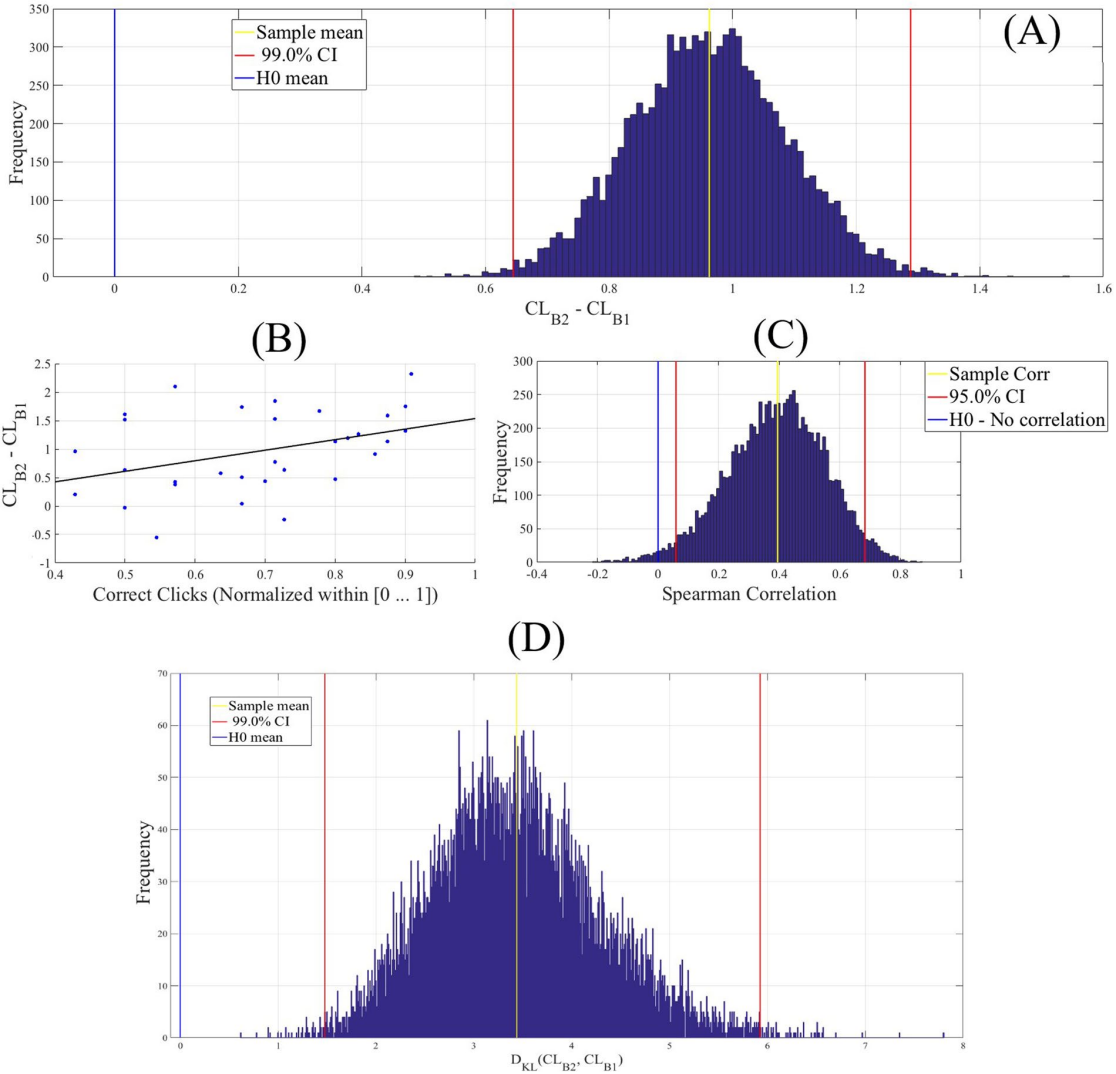
(**A**) One-sample bootstrap test of significance (10,000 simulation runs) at 99.0% confidence interval on the difference between participants’ CLs during one- and two-back WM tasks. In this subplot, the x-axis shows CL_*B*2_ − CL_*B*1_. The blue line marks the null hypothesis *H*0 i.e., non-significant change in CLs during two-back and with respect to one-back WM task. The red lines are the boundaries of the 99.0% confidence interval. The yellow line shows the location of the average CL_*B*2_ − CL_*B*1_ value for 10,000 simulation runs, the red-lines mark the 99.0% confidence interval, and the blue is H0. (**B**) Spearman correlation between participants’ number of correct clicks in response to two-back WM task and the difference between their CLs in one- and two-back WM tasks (i.e. CL_*B*2_ − CL_*B*1_). (**C**) Bootstrap correlation test (10,000 simulation runs) at 95.0% confidence interval in which the observed correlation between CL_*B*2_ − CL_*B*1_ and participant’s correct click during two-back WM task was verified. (**D**) One-sample bootstrap test of significance (10,000 simulation runs) at 99.0% confidence interval on Kullback?Leibler divergence D_*KL*_ between participants’ CLs distribution in two- versus one-back WM tasks. In this subplot, the axis shows the calculated D_*KL*_ between CLs’ distributions in two- (B2) and one-back (B1) (i.e., D_*KL*_(CL_*B*2_, CL_*B*1_)). The yellow line shows the location of the average CL_*B*2_ − CL_*B*1_ value for 10,000 simulation runs, the red-lines mark the 99.0% confidence interval, and the blue is H0 i.e., the non-divergence between the two distributions.

One-sample bootstrap test of significance (10,000 simulation runs) at 99.0% confidence interval (CI) on the difference between participants’ CLs during two-back and one-back WM tasks (i.e., CL_*B*2_ − CL_*B*1_) verified that (Fig. 4(A)) the changes in participants’ CLs during two-back WM task were significantly associated with the cognitive load associated with this WM task period (i.e., CL_*B*2_ − CL_*B*1_ > 0.0) than being the residual effect from their one-back WM task period (M_*CLB*2−*CLB*1_ = 0.96, SD_*CLB*2−*CLB*1_ = 0.71, CI_*CLB*2−*CLB*1_ = [0.74 1.18] where M and SD refer to the mean difference and the standard deviation of such a difference between the two compared states and CI shows the 99.0% confidence interval of their difference).

We also observed a significant correlation between participants’ CL_*B*2_ − CL_*B*1_ values and their correct click during their two-back WM task period (Fig. 4(B), r = 0.39, p = 0.03) which was further supported by their corresponding bootstrap tests (10,000 simulation runs) at 95.0% confidence interval (Fig. 4(C), CI_95.0%_ = [0.05 0.67]).

Finally, the bootstrap test of significance (10,000 simulation runs) at 99.0% confidence interval on the Kullback-Leibler divergence (Fig. 4(D)) between participants’ CLs distribution in two- versus (i.e., D_*KL*_(CL_*B*2_, CL_*B*1_)) identified a significant difference in the distribution of the participants’ CLs in two-back (i.e., B2 in this subplot) and their corresponding CLs during one-back (i.e., B1 in this subplot) WM tasks (M_*DKL*(*B*2, *B*1)_ = 3.44, SD_*DKL*(*B*2, *B*1)_ = 0.87, CI_*DKL*(*B*2, *B*1)_ = [1.48 5.93]). This test ruled out that the observed changes in the participants’ CLs during the two-back were primarily due to the proceeding one-back task (e.g., effect of noise, linear scaling, or affine transformation).

### Experiment 2: Estimation of the perceived difficulty during naturalistic storytelling

The first experiment showed that our proposal for quantification of the cognitive load can significantly discriminate the differential load of the WM tasks on PFC activity. In the second experiment, we verified the ability of our approach in estimation of the individuals’ perceived difficulty of the verbally communicated content in a naturalistic storytelling. We used our *CL*’s formulations and the decision boundary computed with the data used in the second experiment. We also investigated the performance of our approach in four media settings: face-to-face, humanoid, speaker, and video-chat. These media settings allowed us to validate the robustness of our approach. Precisely, the face-to-face setting laid down a reliable basis for verification of our model’s performance: throughout the history, stories have been made and narrated by the people for the people. On the other hand, the speaker and the video-chat verified the utility of our approach in capturing the PFC activation in response to the content of the story than such potential factors as embodiment and novelty effect (in case of the humanoid). Taken together, comparable performance of our model on these media settings in conjunction with its accuracy in case of the humanoid demonstrated its generalizability and therefore effectiveness of the brain-based quantification of the perceived difficulty of the communicated content using PFC pattern of activation.

Contents of Sections 3.2.2 through 3.2.4 are also appeared in Keshmiri *et al*.^55^. For the sake of clarity, we provide their outline in this article as well.

#### Participants

Our participants consisted of twenty three younger adults (fifteen females and eight males, M = 22.39, SD = 2.82). Data from three females were not recorded properly and were discarded. All participants in the first and second experiments were free of neurological and psychiatric disorders and had no history of hearing impairment. All experiments were carried out with written informed consents from all subjects. We used a job-offering site for university students to recruit our participants.

#### Media

They included a humanoid robotic medium, an audio speaker, a video-chat system, and a human. We chose a minimalist teleoperated humanoid called, Telenoid R4^*TM*^ (Telenoid hereafter)^56^. Telenoid is approximately 50.0 cm long and weighs about 3.0 kg. It comes with nine degrees-of-freedom (3 for its eyes, 1 for its mouth, 3 for its neck, and 2 for its arms) and is equipped with an audio speaker on its chest. It is primarily designed to investigate the basic and essential elements of embodiment for the efficient representation and transfer of a humanlike presence. Therefore, its design follows a minimalist anthropomorphic principle to convey a gender-and-age-neutral look-and-feel. In present study, we chose a minimalist anthropomorphic embodiment to eliminate the projection of such physical traits as gender and age onto our robotic medium.

Telenoid conveyed the vocal information of its teleoperator through its speaker. Its motion was generated based on the operator’s voice, using an online speech-driven head motion system^57^. However, its eyes and arms were motionless in this study. We placed Telenoid on a stand approximately 1.40 meters from the participant’s chair to prevent any confounding effect due to tactile interaction (e.g., holding, hugging, etc.). We adjusted this stand to resemble an eye-contact setting between Telenoid and the participant. We maintained the same distance in the case of the other media as well as for the face-to-face setting. In face-to-face condition, we adjusted the storyteller’s seat to maintain eye-contact with the participant. For the video-chat, we adjusted its placeholder in such a way that the storyteller’s appearance on the screen resembled an eye-contact setting. In the speaker setting, we placed the video-chat screen in front of the participant (like in the video-chat condition) and placed the speaker behind its screen.

We used the same audio device in the speaker and video-chat settings to prevent any confounding effect due to audio quality. We used the same recorded voice of a woman, who was naive to the purpose of this study, in speaker, video-chat, and Telenoid. These recordings took place in a single session in which we recorded her voice and video while telling stories. In Telenoid setting, we played back the same prerecorded voice for the speaker through the audio speaker on its chest. In face-to-face setting, the same woman read the stories to the participants.

We asked our female storyteller to stay as neutral as possible while reading these stories. However, we are unable to confirm the absence of any difference in emotional impact of the stories’ content on her during the face-to-face or the voice/video recordings.

#### Paradigm

It consisted of three-minute storytelling sessions in which a woman narrated three-minute stories from Greek mythology through three kinds of communication media: an audio speaker, a video-chat system, and Telenoid. As a control setting, we included the face-to-face scenario in which we told these stories to the participants in-person. Every individual participated in all four storytelling sessions. We also controlled the field of view of the participants within the same spatial limit by placing their seat in a cubicle in all these sessions (height = 130.0 cm, width = 173.0 cm, depth = 210.0 cm) which further enabled us to prevent the potential confounding effect of visual distraction.

#### Procedure

It started with collecting the written informed consents from the participants which was then followed by a male experimenter explaining the experimental procedure to them. In this step, the experimenter explained to the participants that each storytelling session was approximately three minutes in length and that each session would start with a one-minute resting period during which they were required to sit still with their eyes closed. He then briefed them about the content of stories and instructed them to stay focused on these stories’ content as much as possible. After leading the participants to the experimental room, the experimenter helped them be seated and ensured the proper adjustment of the medium (or helped the storyteller get in her proper position during the face-to-face setting) and began the experimental session. In every session, after the experimenter acquired the one-minute resting data, he asked the participants to open their eyes and get ready for the start of the story which was then immediately followed by the story being told. During the face-to-face setting, we asked the storyteller to maintain as much eye-contacts with the participants as possible. We also used the Tobii Eye Tracker 4C controller which has a sampling rate of 90.0 Hz to acquire the participants’ eye-movement data. Tobii was placed at approximately 80.0 cm from the participant’s seat and 30.0 cm above the ground. To avoid any interference between the eye-tracker and the fNIRS devices, we placed it between the stand of the media and the chair of participants.

Once a storytelling session was over, the participants filled in a questionnaire that asked them how difficult they thought the story content was. We used an 8-point scale questionnaire in which “1” meant “not difficult at all” and “8” denoted “very difficult” story content. We gave our participants a one-minute rest period prior to the commencement of each of the storytelling sessions and asked them to keep their eyes closed. In this period, we prepared the setting for the next storytelling session. We video-recorded all the activities throughout the experiment.

Every individual participated in each of the four storytelling sessions. We kept the content of the stories and their orders in these sessions intact (i.e., the first story was always the same for all participants and regardless of the medium) while randomizing the order of the media. Each experiment took about 90 minutes for each participant.

#### Analysis

We used the decision boundary 𝔻 that was determined based on one- and two-back WM tasks’ *CL*s in the second experiment during the realtime storytelling experiment.

We followed the same procedure as in the first experiment for data acquisition and processing. At every estimation step in current implementation, our model calculated the *CL* of the current PFC activity time series (per participant per medium). At the end of the storytelling session, our model computed the median of these computed *CL*s and determined whether it was above or below the decision boundary 𝔻. Subsequently, it marked the individual’s perception of the story content as “difficult” if this median was above the decision boundary. Otherwise, it marked it as “easy.”

We also used these medians per participant along with their self-assessed responses to the difficulty of the story’s content per medium to determine our model’s true positive (tp), true negative (tn), false positive (fp), and false negative (fn). Concretely, we considered self-assessed responses 1 through 4 and 5 though 8 to represent “easy” and “difficult” content, respectively. We then evaluated our model’s estimate as a “Hit” if Mdn_*CLparticipant*_ was above the decision boundary 𝔻 and the self-assessed response ≥5 or if Mdn_*CLparticipant*_ was below the decision boundary 𝔻 and the self-assessed response ≤4 (i.e., if they matched). Otherwise, we evaluated its estimate as a “Miss.” Every “Hit” by our model contributed to a “tp” (i.e., Mdn_*CLparticipant*_ was above the decision boundary 𝔻 and the self-assessed response ≥5) or a “tn” (i.e., Mdn_*CLparticipant*_ was below the decision boundary 𝔻 and the self-assessed response ≤4). On other hand, we considered the model’s estimate as a “fp” if Mdn_*CLparticipant*_ was above the decision boundary 𝔻 and the participant’s self-assessed response ≤4. Similarly, we considered it as a “fn” if Mdn_*CLparticipant*_ was below the decision boundary 𝔻 and the participant’s self-assessed response ≥5. We used these tp, tn, fp, and fn values to calculate the confusion matrix, the accuracy, the precision, the recall, and the F1-score of our model in each of the media settings.

Last, we computed the Spearman correlation between participants’ self-assessed responses to difficulty of story content and their CLs. In case of all results, we reported the results for the left-hemispheric PFC in the manuscript. We provided the results for the right-hemispheric PFC in SM 2.2.

#### Results

**Figure 5:**
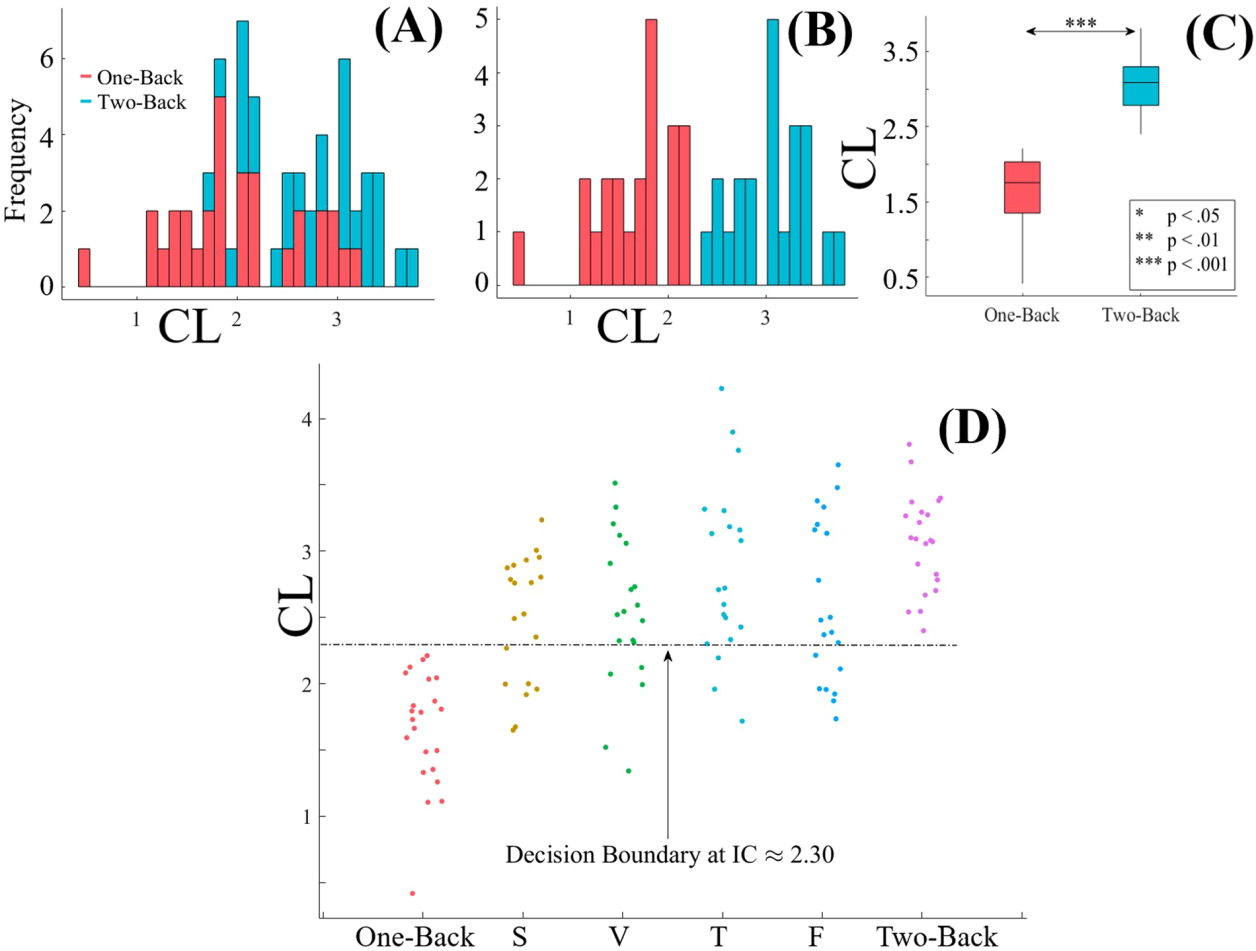
(**A**) One- (red) and two-back (blue) *CL*s prior to application of Algorithm 1. (**B**) Non-overlapping *CL*s of one- (red) and two-back (blue) WM tasks. (**C**) Wilcoxon rank sum between refined *CL*s of one- and two-back WM tasks. Asterisks mark their differential significance. (**D**) Refined one- and two-back *CL*s along with the medians of the *CL*s of the participants’ PFC activation during storytelling experiment (as computed by our proposed model) in speaker (S), video-chat (V), Telenoid (T), and face-to-face (**F**) media settings. The decision boundary 𝔻 at *CL* ≈ 2.30 is shown in this subplot. The CLs associated with one- and two-back WM tasks are also presented in this subplot to better visualize the correspondence between the decision boundary 𝔻 and these CLs distributions.

Figure 5(A) shows the distribution of one- (red) and two-back (blue) *CL*s prior to the application of Algorithm 1. Figure 5(B) plots their resulting non-overlapping *CL*s after the application of this algorithm (nine *CL*s per task discarded in total). Wilcoxon rank sum indicated that (Fig. 5(C)) the significant difference between one- and two-back CLs after the refinement step was preserved (p < 0.001, W(42) = 5.67, r = 0.85, M_*one*−*back*_ = 1.65, SD_*one*−*back*_ = 0.43, M_*two*−*back*_ = 3.07, SD_*two*−*back*_ = 0.37).

Figure 5(D) shows the median of the participants’ *CL*s during the realtime storytelling experiment in speaker (S), video-chat (V), Telenoid (T), and face-to-face (F) media settings. These *CL*s are presented next to one- and two-back *CL*s for better visualization of their distribution with respect to these WM tasks’ *CL*s. The decision boundary 𝔻 by Algorithm 1 𝔻 at *CL* ≈ 2.30 is shown in this subplot.

Our model was able to predict the participants’ perceived difficulty of the story content with 80.0% prediction accuracy in the speaker setting. In addition, its accuracy was 85.0% in case of the video-chat and Telenoid media. Last, it predicted their perceived difficulty of the story content with 90.0% accuracy during the face-to-face setting. Table 2 summarizes the performance statistics of our model in these media settings during the realtime storytelling experiment.

**Table 2:** Proposed model’s accuracy, precision, recall, and F1-score during the realtime storytelling experiment.

Media Setting	Accuracy (%)	Precision	Recall	F1-score
S	80.0	0.92	0.80	0.86
V	85.0	0.93	0.88	0.90
T	85.0	0.88	0.94	0.91
F	90.0	1.0	0.87	0.93

**Figure 6:**
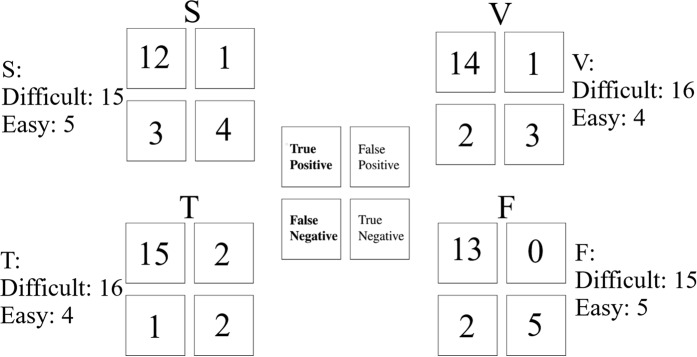
Confusion matrices of our model during the storytelling experiment for speaker (S), video-chat (V), Telenoid (T), and face-to-face (F) media. In this figure, “Difficult” and “Easy” represent the number of participants whose self-assessed responses indicated “difficult” or “easy” story content. Results of the participants’ self-assessment responses to difficulty of the story content identified that fifteen and five participants considered the story content difficult and easy in the speaker and the face-to-face. These numbers were sixteen and four in the video-chat and the Telenoid settings.

Figure 6 shows the confusion matrices of our model during the storytelling experiment in speaker (S), video-chat (V), Telenoid (T), and face-to-face (F) media settings. We observed that our model was stronger in differentiating the participants’ PFC activation in response to perceived difficulty of the story content in case of the Telenoid (Fig. 6T, True Positive block). Similarly, it differentiated best the content that was perceived “easy” by the participants in case of the face-to-face setting (Fig. 6F, True Negative block). On the other hand, it did slightly worse for estimation of the perceived difficulty of the story content in case of speaker (Fig. 6S, False Negative). Whereas it misidentified two “easy” cases as “difficult” content in case of the Telenoid (Fig. 6T, False Positive block), such a misestimation was one in case of the speaker (Fig. 6S, False Positive block) and the video-chat (Fig. 6V, False Positive block).

These observations were further supported by the precision, recall, and F1-score of the model’s performance. Table 2 indicates that whereas our model’s performance obtained its highest precision in the face-to-face setting that was followed by the video-chat and speaker, its recall was highest in the case of Telenoid. Interestingly, this table also reveals a direct correspondence between the physical embodiment of the medium and the performance of our model. Specifically, we observed that face-to-face and Telenoid were associated with the first and the second highest F1-score values among the four media settings. We also observed that our model achieved its highest accuracy in the face-to-face setting which was followed by its higher accuracy in the case of Telenoid and the video-chat while the speaker setting was associated with lowest model accuracy among the four media settings.

**Figure 7:**
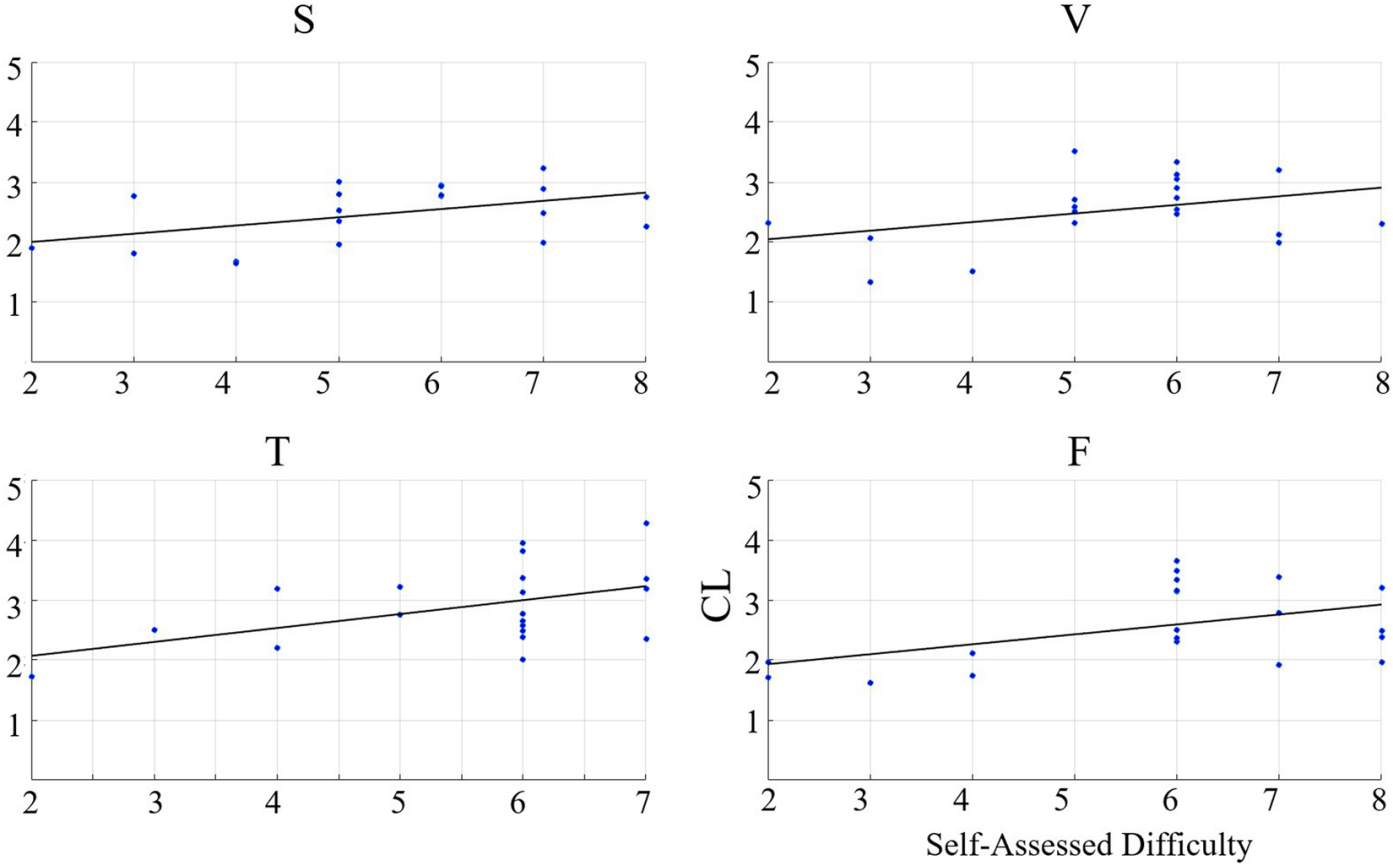
Left PFC: Spearman correlation between participants’ self-assessed difficulty of story content and their CLs in speaker (S), video-chat (V), Telenoid (T), and face-to-face (F) settings.

Last, we observed significant correlations (Fig. 7) between the participants’ self-assessed responses to difficulty of the story content and their CLs in speaker (r = 0.50, p < 0.03, uncorrected), video-chat (r = 0.43, p < 0.05, uncorrected), Telenoid (r = 0.48, p < 0.05, uncorrected), and face-to-face (r = 0.47, p < 0.05, uncorrected) settings.

## Discussion

In this article, we presented a novel information-theoretic approach to quantification of the cognitive loads associated with the human subjects’ PFC activity. Our choice of information theory^41^ for modeling of the PFC pattern of activity was motivated by the neuroscientific findings that have provided compelling empirical^30–32^ and theoretical^58,59^ evidence that emphasize the crucial role of signal variability in the quantification of the brain activity. This, in turn, has resulted in emerging viewpoints that identify the role of entropy in brain functioning^60^. For instance, our quantification of the induced cognitive load on PFC in terms of the interplay between mutual information (i.e., convergence) and Kullback-Leibler divergence is motivated by a new thesis on the entropic nature of the brain^61^ that itself is based on findings that identify the functioning of the brain near criticality^62,63^.

We showed that the use of this approach (in conjunction with a decision boundary that corresponded to the refined cognitive loads induced by the one- and two-back auditory tasks) was successful in predicting the individuals’ perceived difficulty of communicated content in a storytelling scenario. Our results indicated that our model maintained a significantly above average (50.0% chance level in case of binary classification) prediction accuracy with a comparable performance in face-to-face, humanoid, speaker, and video-chat media settings. This verified that the quantified participants’ PFC activation in the form of *CL* was primarily due to the effect of communicated content than such confounders as choice of the medium. This observation was also supported by the comparative analysis of the participants’ self-assessment responses in different media settings (SM Section 3). More importantly, our model was able to estimate the effect of stories’ difficulty on individuals’ PFC activity bilaterally which was in line with the neuroscientific findings on bilateral effect of stories on human subjects’ PFC^13–15^.

Previous findings have shown that certain patterns of brain activity can provide means to uncover the subjective contents that might at least partly be shared among individuals^64–66^. Our results extended these findings by demonstrating that such potential shared spaces can be utilized for decoding of the individuals’ PFC activity to predict their perceived difficulty of the narrated stories. From a broader perspective, our approach was related to research that focuses on the application of machine learning^67^ and other computational paradigms^68^ for decoding of the brain activity in response to stimuli.

An interesting observation during the storytelling experiment was the incremental improvement of the prediction accuracy of our model from speaker (i.e., total absence of storyteller) to face-to-face settings. In addition, we observed that our model achieved its highest correctly predicted easy (i.e., true negative) and difficult (i.e., true positive) contents in the face-to-face and the humanoid settings, respectively. These results may suggest the potential benefit of the physical embodiment on quantification of the human subjects’ PFC activation in response to verbally communicated contents. However, further investigation of the extent of such an effect is necessary prior to drawing any conclusion on this possibility.

Our results contribute to such socially assistive robotics^1,69^ scenarios as child education and elderly care. For instance, our model can enable these media to determine whether their level of interaction (e.g., socialization^2^, reading and comprehension^3^) is exceeding the comfort level of children, thereby allowing for modulation of their communicated contents and/or behavioural interaction. Our model can also further enhance the use of these media in robot-assistive cognitive training of the older people^70^. For example, robots equipped with our model can be used during cognitive training of older people in elderly care facilities to monitor these individuals’ brain activity during their training, thereby allowing the cognitive trainers to determine the older people’s level of comfort in continuing their training session^4,5^.

Our model’s performance during the storytelling experiment that was in line with the neuroscientific findings on the effect of story on human subjects’ PFC activation^13–15^ indicated a promising first step toward the use of brain information for quantification of one of the basic component of the human mental state: perceived difficulty of verbally communicated contents. These results benefit such HRI paradigms as interactive learning^38^ by allowing these algorithms to utilize the individuals’ patterns of brain activity as real-time neurological feedbacks to refine their interaction strategy. In a broader perspective, our findings can benefit the research on PFC activation during social cognition^12,13^ and its involvement in story-based ToM analyses^71^. More importantly, the ability of the robots to estimate their human companions’ perceived difficulty of their verbal communication can contribute to formal analysis of a robotic ToM^39^ through critical investigation of its implications in humans’ neurological responses while interacting with these agents.

### Limitations and future direction

Although our model indicated a promising first step toward quantification of the human subjects’ PFC activation in response to difficulty of verbally communicated content, a larger human sample is necessary for an informed conclusion on its utility. Moreover, our participants were limited to the younger adults. Therefore, it is necessary to investigate the performance of our model in other age groups (e.g., kids, adolescents, older people) to verify that its performance is unaffected by this factor.

Our analyses indicated that our model was able to quantify the PFC activity in response to one- and two-back auditory tasks differentially, thereby allowing for their classification with a high accuracy. An important issue in this regard that demands further investigation is the order by which the participants performed these WM tasks. In particular, in our study every individual first performed the one-back which was then followed by the two-back task. Although our further analyses identified that the CLs associated with two-back WM task was significantly due to the effect of this task than a residual effect of one-back task. it is crucial for the future studies to investigate whether counter-balancing the order of these task may impose any impact on the performance of the proposed approach.

We also observed that participants’ self-assessed difficulty of stories correlated with change in their PFC activity. This highlighted the potential presence of a direct correspondence between individuals’ subjective evaluation of the communicated contents (e.g., stories) and the effect of such contents on their PFC information processing. On the other hand, whereas we observed that such correlations were bilateral in the case of speaker, Telenoid, and face-to-face settings, it was only present in left-hemispheric PFC in the case of video-chat. This differed from our prediction results in which we observed that our model was able to significantly predict the difficulty of story content in all media settings, that such significantly above chance predictions were present bilaterally in the case of all media, and that the increase of such predictions were incremental in the level of embodiment. Therefore, the future research is necessary to further investigate the source of such a difference between correlation and prediction analyses.

Moreover, our experimental setting was limited to a storytelling scenario in which participants listened to a verbally communicated content without any requirement for their response. Therefore, it is crucial to examine the performance of our model in such verbal communication scenarios as conversation to ensure its performance is unaffected by any potential PFC activation induced by such bidirectional verbal communications.

We used the participants’ self-assessed responses to difficulty of stories to investigate the accuracy of our model. This constrained our validation procedure in that we were forced to wait until the storytelling ended and therefore were not able to examine its performance finer time scales such as minute-prediction-cycle or shorter. This constraint was imposed by the fact any finer-scale prediction cycle inevitably needs us to interrupt of the individuals’s listening to the stories to inquire about their subjective feeling of the story’s difficulty, thereby disrupting their thoughts as well as the course of verbal communication. Therefore, future research to look into alternative strategies to acquire individuals’ self-assessment during the communication is necessary to draw a more informed conclusion on performance of our model.

We observed an incremental prediction accuracy from the speaker to face-to-face media settings. We also observed that our model achieved its highest correctly predicted easy (i.e., true negative) and difficult (i.e., true positive) contents in the face-to-face and the humanoid settings, respectively. This suggested a potential positive effect of embodiment on quantification of the PFC activation during verbal communication. However, present study did not include other types of physically embodied media (e.g., mechanical looking robots, pet robots, etc.). Therefore, it is necessary to determine the correspondence between the media embodiment and the observed incremental accuracy of our model. It is also important to verify whether different embodiments can induce differential impact on this incremental pattern of accuracy.

Our approach is based on information theory. Integral to this formalism is the Data Processing Inequality (DPI) [^41^, p. 34, Theorem 2.8.1] which states that the more the data manipulation (e.g., data pre/processing steps) the more the loss of information. Therefore, it was crucial for us to apply the pre/processing steps as efficiently as possible. In this regard, Cooper *et al*.^52^ showed that PCA-based motion correction algorithm yielded a significant reduction in the mean-squared error (MSE) and a significant increase in contrast-to-noise ratio (CNR) in comparison with no correction and/or the process of rejecting the motion-contaminated trials. Considering the fNIRS more immunity to body movement as compared with other neuroimaging techniques^72^, the minimal physical activity in our experimental settings (e.g., clicking mouse in n-back), and the recent findings that indicated that the measured PFC activity during listening to stories was not affected by such behavioural responses as eye-movement^55^, we found the application of PCA sufficient for addressing both SBF attenuation and potential minimal motion artefacts correction, thereby adhering with DPI principle as much as possible. However, the future research requires to take into consideration the effectiveness of our methodology in the use of PCA in scenarios in which substantially more physical activity and/or embodied interaction is expected, thereby examining the utility of other approaches for motion artefacts correction^52^.

## Supplementary information

Supplementary Materials File
