# Supplementary material for: Information Content of Prefrontal Cortex Activity Quantifies the Difficulty of Narrated Stories
Source: Sci Rep. 2019 Nov 29;9:17959. doi: 10.1038/s41598-019-54280-1 (PMC6884437; doi:10.1038/s41598-019-54280-1)
Supplement: Supplementary file 1 — Supplementary Materials File [file 41598_2019_54280_MOESM1_ESM.pdf]

Information Content of Prefrontal Cortex Activity Quantifies the Difficulty of Narrated Stories

Soheil Keshmiri[1,*], Hidenobu Sumioka[1], Ryuji Yamazaki[2], Masahiro Shiomi[1], and Hiroshi Ishiguro[1,3]

[1]Advanced Telecommunications Research Institute International (ATR), Kyoto, Japan
[2]Symbiotic Intelligent Systems Research Center, Institute for Open and Transdisciplinary Research Initiatives, Osaka University, Japan
[3]Graduate School of Engineering Science, Osaka University, Japan
[*]soheil@atr.jp

ABSTRACT

Section 1 presents the results of the left-hemispheric PFC activation during one- and two-back WM tasks using average PFC activation. In Sections 2.1.1 and 2.1.2 we demonstrate the effectiveness of our approach by comparing its performance on quantification of the right-hemispheric PFC activation during one- and two-back WM tasks against the scenario in which average PFC activation is used. In Section 2.2, we present its performance on the right-hemispheric PFC during realtime storytelling experiment. Section 3 presents the Kruskal-Wallis analysis of the participants' self-assessment in speaker, video-chat, Telenoid, and in-person media settings during the storytelling experiment.

1 Quantification of the Left-Hemispheric PFC Activation During N-Back WM Task Using Average PFC Activation

Wilcoxon rank sum (Figure 1 (A)) indicated that the difference between participants' average PFC activation in one- and two-back WM tasks was non-significant (p = .74, W(60) = .34, r = .04, $M_{one-back}$ = .01, $SD_{one-back}$ = .03, $M_{two-back}$ = .02, $SD_{two-back}$ = .05). Use of the average PFC activation resulted in 41.94% prediction accuracy between one- and two-back WM tasks. Table 1 summarizes these results. Figure 1 (B) shows the one-to-one correspondence between participants' average PFC activation in one- and two-back WM tasks.

We observed (Figure 1 (B)) no correlation between participants' average PFC activation and their number of correct clicks in response to one-back (r = .10, p = .60) WM task. Similarly, we found (Figure 1 (C)) no correlation between participants' average PFC activation and their correct clicks in two-back WM task (r = .21, p = .25). Last, we found (Figure 1 (D)) no correlation between their average PFC activation in one- and two-back WM tasks (r = .19, p = .30).

2 Right-Hemispheric PFC Activation

2.1 N-Back Experiment

2.1.1 Quantification of the Right-Hemispheric PFC Activation During N-Back WM Task Using the Proposed Model

Wilcoxon rank sum (Figure 2 (A)) identified a significant difference between the participants' *CL*s in one- and two-back WM tasks (p < .001, W(60) = 3.59, r = .46, $M_{one-back}$ = 1.52, $SD_{one-back}$ = .59, $M_{two-back}$ = 2.18, $SD_{two-back}$ = .64). Our model achieved an 87.10% prediction accuracy for classification of these tasks. Table 2 first row entry summarizes these results.

Table 1. Wilcoxon rank sum along with the mean and standard deviation of the one- (M_1 and SD_1) and two-back (M_2 and SD_2) WM tasks using average PFC activation (Mean). r is the effect size. Mdn stands for Median. We checked the mean and the median of the average PFC activation and noted that the use of median yielded a higher accuracy. Therefore, we reported the median of the participants' average PFC activation than their mean for prediction accuracy in this case as well.

	p	W(60)	r	M_1	SD_1	M_2	SD_2	$Mdn_2 > Mdn_1 (\%)$
Mean	= .74	.34	.04	.01	.03	.02	.05	41.94

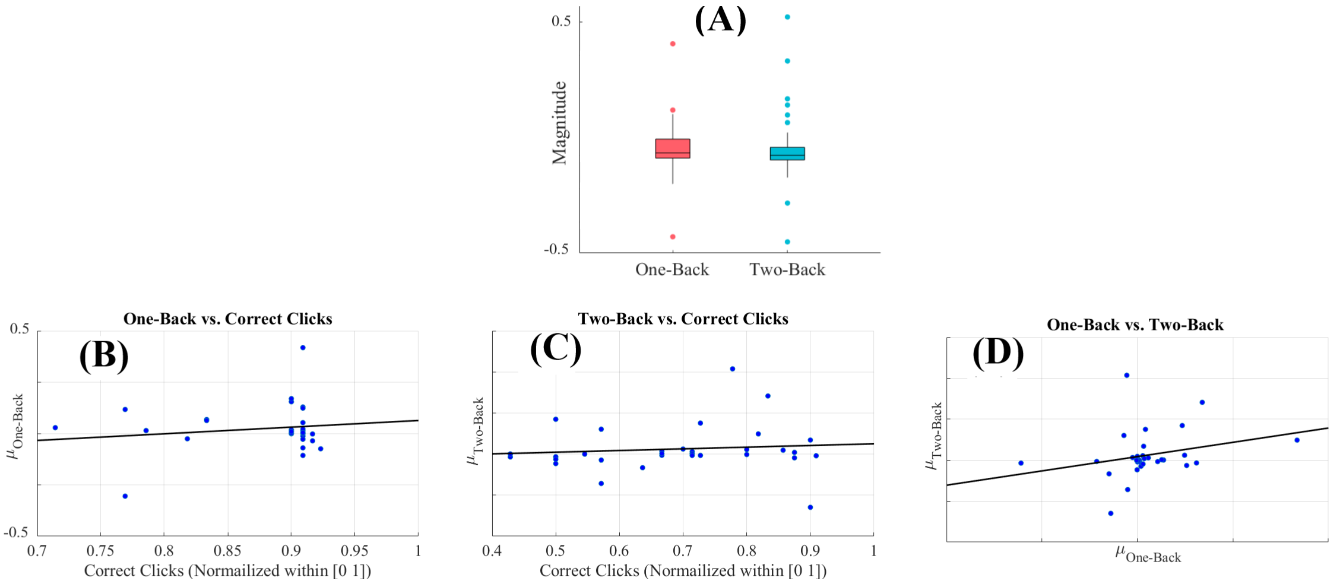

Figure 1. (A) Wilcoxon rank sum between average PFC activation in one- and two-back WM tasks. (B) Spearman correlation between average PFC activation and the number of participants' correct clicks in one-back WM task. (C) Spearman correlation between average PFC activation and the number of participants' correct clicks in response to two-back WM task. (D) Spearman correlation between average PFC activation in one- and two-back WM tasks. In (B) and (C) correct clicks are normalized within [0, ..., 1] interval.

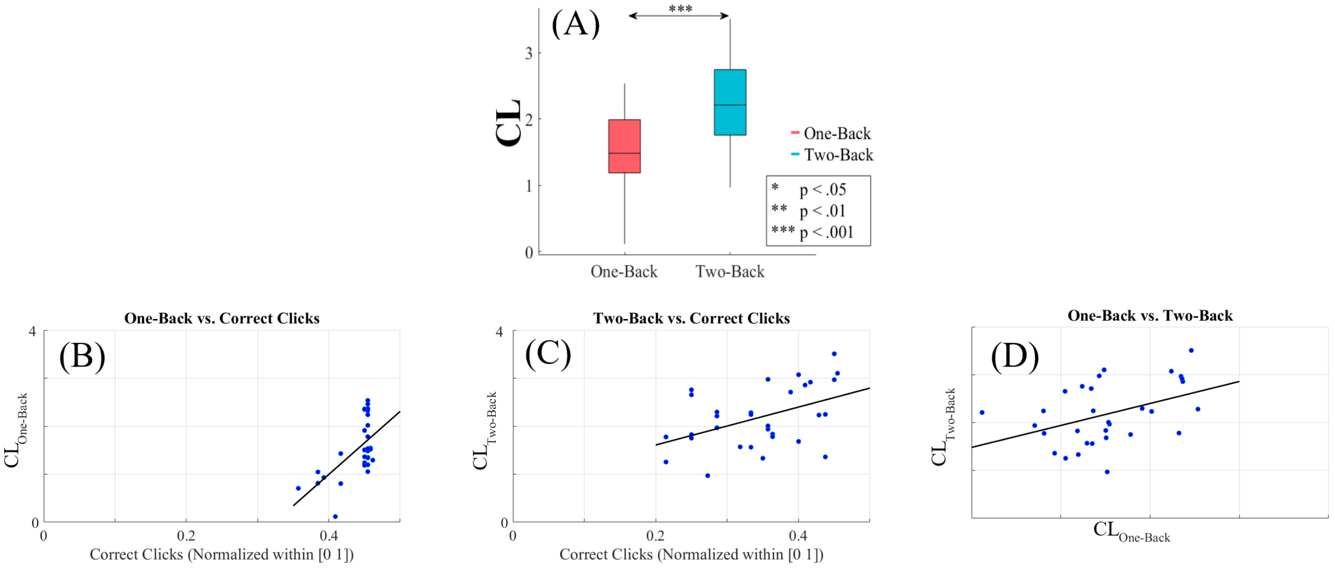

Figure 2. Right-Hemispheric PFC: (A) Wilcoxon rank sum between participants' *CL*s in one- and two-back WM tasks. Asterisks indicate the significant difference between these *CL*s. (B) Spearman correlation between participants' *CL*s and the number of their correct clicks in one-back WM task. (C) Spearman correlation between participants' *CL*s and the number of their correct clicks in response to two-back WM task. (D) Spearman correlation between participants' *CL*s in one- and two-back WM tasks. In (B) and (C) correct clicks are normalized within [0, ..., 1] interval.

We found a significant correlation between participants' *CL*s and their number of correct clicks in response to one-back (Figure 2 (B)) WM task (r = .60, $p < .001$, M_{Clicks} = .88, SD_{Clicks} = .05). Similarly, this correlation was significant in two-back (Figure 2 (C)) WM task (r = .49, $p < .01$, M_{Clicks} = .69, SD_{Clicks} = .15). Last, we observed (Figure 2 (D)) a significant correlation between participants' *CL*s in one- and two-back WM tasks (r = .42, $p < .03$).

One-sample bootstrap test of significance (10,000 simulation runs) at 99.0% confidence interval (CI) on the difference

Table 2. Wilcoxon rank sum along with the mean and standard deviation of the one- (M_1 and SD_1) and two-back (M_2 and SD_2) WM tasks' CLs vs. average right-hemispheric PFC activation (Mean). r is the effect size. Mdn stands for Median. We calculated the accuracy in differentiating the participants's PFC activation in response to one- and two-back WM tasks using the median of the adapted measure. We checked the mean and the median of the average PFC activation and noted that the use of median yielded a higher accuracy. Therefore, we reported the median of the participants' average PFC activation than their mean for prediction accuracy in this case as well.

	p	W(60)	r	M_1	SD_1	M_2	SD_2	$Mdn_2 > Mdn_1 (\%)$
CL	$< .001$	3.59	.46	1.52	.59	2.18	.64	87.10
Mean	$= .60$	.52	.07	.01	.03	.02	.01	38.71

between participants' CLs during two-back and one-back WM tasks (i.e., CL_{B2} - CL_{B1}) verified that (Figure 3 (A)) the changes in participants' CLs during two-back WM task were significantly associated with the cognitive load associated with this WM task period (i.e., CL_{B2} - $CL_{B1} > 0.0$) than being the residual effect from their one-back WM task period ($M_{CL_{B2}-CL_{B1}}$ = 1.12, $SD_{CL_{B2}-CL_{B1}}$ = .70, $CI_{CL_{B2}-CL_{B1}}$ = [.92 1.33] where M and SD refer to the mean difference and the standard deviation of such a difference between the two compared states and CI shows the 99.0% confidence interval of their difference).

We also observed a significant correlation between participants' CL_{B2} - CL_{B1} values and their correct click during their two-back WM task period (Figure 3 (B), r = .38, p = .03) which was further supported by their corresponding bootstrap tests (10,000 simulation runs) at 95.0% confidence interval (Figure 3 (C), $CI_{95.0\%}$ = [.05 .66]).

Finally, the bootstrap test of significance (10,000 simulation runs) at 99.0% confidence interval on the Kullback-Leibler divergence (Figure 3 (D)) between participants' CLs distribution in two- versus (i.e., $D_{KL}(CL_{B2}, CL_{B1})$) identified a significant difference in the distribution of the participants' CLs in two-back (i.e., B2 in this subplot) and their corresponding CLs during one-back (i.e., B1 in this subplot) WM tasks ($M_{D_{KL}(B2,B1)}$ = 3.90, $SD_{D_{KL}(B2,B1)}$ = .94, $CI_{D_{KL}(B2,B1)}$ = [1.86 6.78]). This test ruled out that the observed changes in the participants' CLs during the two-back were primarily due to the proceeding one-back task (e.g., effect of noise, linear scaling, or affine transformation).

2.1.2 Quantification of the Right-Hemispheric PFC Activation During N-Back WM Task Using Average PFC Activation

Wilcoxon rank sum (Figure 4 (A)) indicated that the difference between participants' average PFC activation in one- and two-back WM tasks was non-significant (p = .60, W(60) = .52, r = .07, $M_{one-back}$ = .01, $SD_{one-back}$ = .03, $M_{two-back}$ = .02, $SD_{two-back}$ = .01). Use of the average PFC activation resulted in 38.71% prediction accuracy between one- and two-back WM tasks. Table 2 second row entry summarizes these results.

We observed (Figure 4 (B)) no correlation between participants' average PFC activation and their number of correct clicks in response to one-back (r = .15, p = .41) WM task. Similarly, we found (Figure 4 (C)) no correlation between participants' average PFC activation and their correct clicks in two-back WM task (r = .10, p = .58). Last, we found (Figure 4 (D)) no correlation between their average PFC activation in one- and two-back WM tasks (r = .26, p = .15).

2.2 Storytelling Experiment

Figure 5 (A) shows the distribution of one- (red) and two-back (blue) right-hemispheric CLs prior to the application of Algorithm 1. Figure 5 (B) plots their resulting non-overlapping CLs after the application of this algorithm (nine CLs per task discarded in total). Wilcoxon rank sum indicated that (Figure 5 (C)) the significant difference between one- and two-back CLs after the refinement step was preserved (p $<$ .001, W(42) = 5.28, r = .84, $M_{one-back}$ = 1.27, $SD_{one-back}$ = .41, $M_{two-back}$ = 2.69, $SD_{two-back}$ = .40).

Figure 5 (D) shows the median of the participants' right-hemispheric CLs during the realtime storytelling experiment in speaker (S), video-chat (V), Telenoid (T), and in-person (F) media settings. These CLs are presented next to one- and two-back CLs for better visualization of their distribution with respect to these WM tasks' CLs. The decision boundary $\mathbb{D}$ at $CL \approx 2.10$ is shown in this subplot. This boundary was at $CL \approx 2.30$ for the left hemisphere.

Our model was able to predict the participants' perceived difficulty of the story content with 80.0% prediction accuracy in the speaker and video-chatting setting. In addition, its accuracy was 90.0% in case of Telenoid and in-person settings. Table 3 summarizes the performance statistics of our model in these media settings during the realtime storytelling experiment.

Figure 6 shows the confusion matrices of our model during the storytelling experiment in speaker (S), video-chat (V), Telenoid (T), and in-person (F) media settings. We observed that our model was stronger in differentiating the participants' PFC activation in response to perceived difficulty of the story content in case of the Telenoid (Figure 6 T, True Positive block). Similarly, it differentiated best the content that was perceived "easy" by the participants in case of the in-person setting (Figure 6 F, True Negative block). On the other hand, it did slightly worse for estimation of the perceived difficulty of the story content in

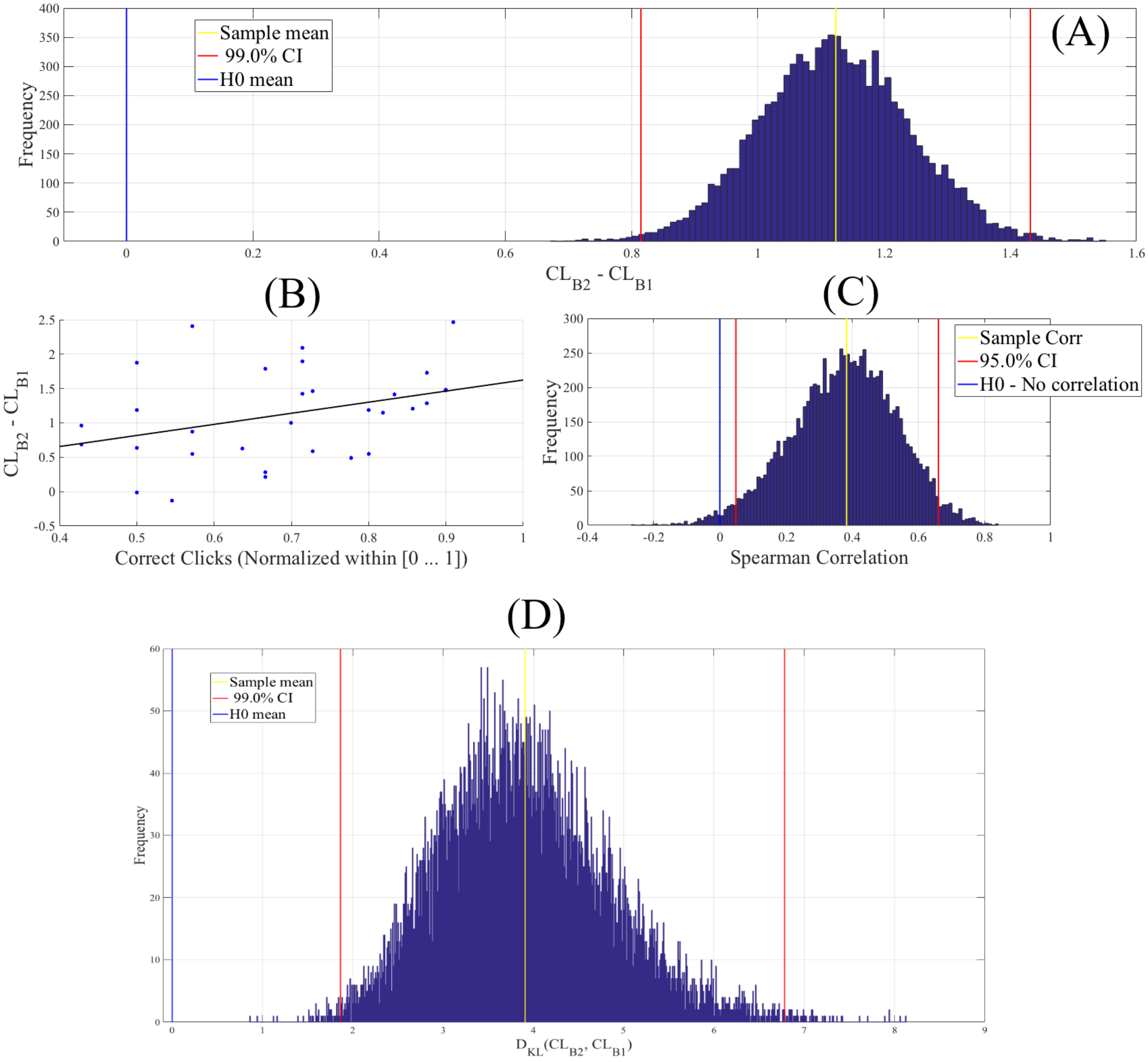

Figure 3. (A) One-sample bootstrap test of significance (10,000 simulation runs) at 99.0% confidence interval on the difference between participants' CLs during one- and two-back WM tasks. In this subplot, the x-axis shows CL_{B2} - CL_{B1}. The blue line marks the null hypothesis $H0$ i.e., non-significant change in CLs during two-back and with respect to one-back WM task. The red lines are the boundaries of the 99.0% confidence interval. The yellow line shows the location of the average CL_{B2} - CL_{B1} value for 10,000 simulation runs. (B) Spearman correlation between participants' number of correct clicks in response to two-back WM task and the difference between their CLs in one- and two-back WM tasks (i.e. CL_{B2} - CL_{B1}). (C) Bootstrap correlation test (10,000 simulation runs) at 95.0% confidence interval in which the observed correlation between CL_{B2} - CL_{B1} and participant's correct click during two-back WM task was verified. (D) One-sample bootstrap test of significance (10,000 simulation runs) at 99.0% confidence interval on Kullback?Leibler divergence D_{KL} between participants' CLs distribution in two- versus one-back WM tasks. In this subplot, the axis shows the calculated D_{KL} between CLs' distributions in two- (B2) and one-back (B1) (i.e., $D_{KL}(CL_{B2}, CL_{B1})$). The yellow line shows the location of the average CL_{B2} - CL_{B1} value for 10,000 simulation runs, the red-lines mark the 99.0% confidence interval, and the blue is H0 i.e., the non-divergence between the two distributions.

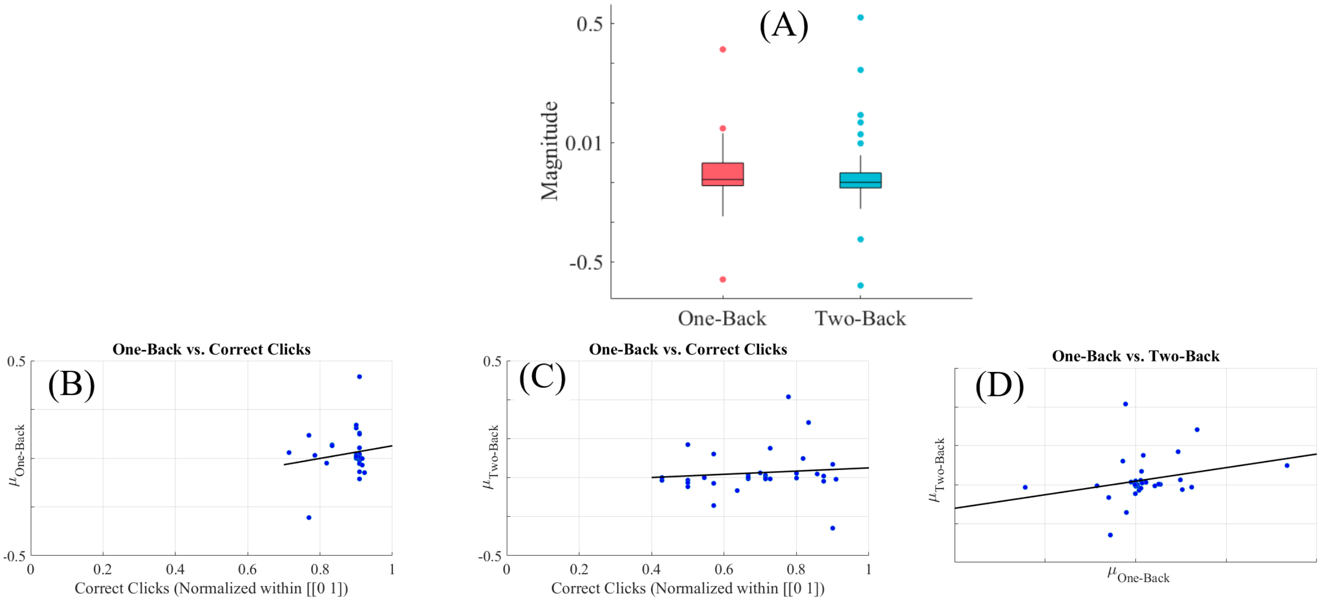

Figure 4. Average Right-Hemispheric PFC Activation: (A) Wilcoxon rank sum between average PFC activation in one- and two-back WM tasks. (B) Spearman correlation between average PFC activation and the number of participants' correct clicks in one-back WM task. (C) Spearman correlation between average PFC activation and the number of participants' correct clicks in response to two-back WM task. (D) Spearman correlation between average PFC activation in one- and two-back WM tasks. In (B) and (C) correct clicks are normalized within $[0, \ldots, 1]$ interval.

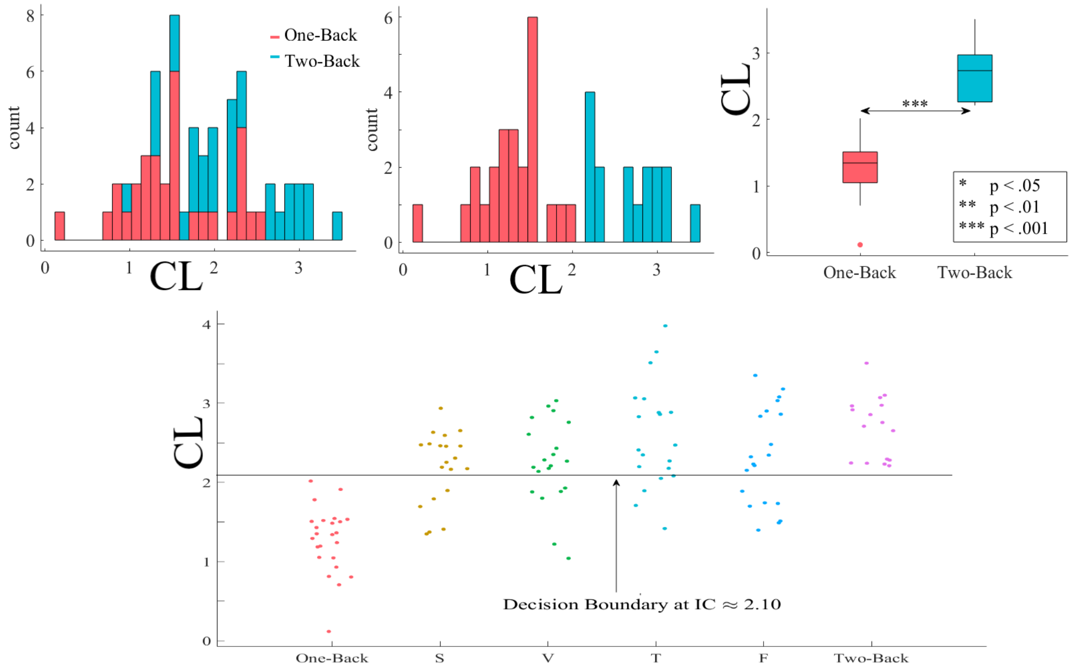

Figure 5. (A) One- (red) and two-back (blue) CLs of right-hemispheric PFC prior to application of Algorithm 1. (B) Refined CLs of one- (red) and two-back (blue) WM tasks. (C) Wilcoxon rank sum between refined CLs of one- and two-back WM tasks. Asterisks mark their differential significance. (D) Refined one- and two-back CLs along with the medians of the participants' right-hemispheric PFC activation during storytelling experiment (as computed by the proposed model) in speaker (S), video-chat (V), Telenoid (T), and in-person (F) media settings. Decision boundary $\mathbb{D}$ at $CL \approx 2.10$ is shown in this subplot. This value was $CL \approx 2.30$ in case of left-hemispheric PFC.

Table 3. Proposed model's accuracy, precision, recall, and F1-score during the realtime storytelling experiment.

Media Setting	Accuracy (%)	Precision	Recall	F1-score
S	80.0	.87	.87	.87
V	80.0	.93	.81	.87
T	90.0	.94	.94	.94
F	90.0	1.0	.87	.93

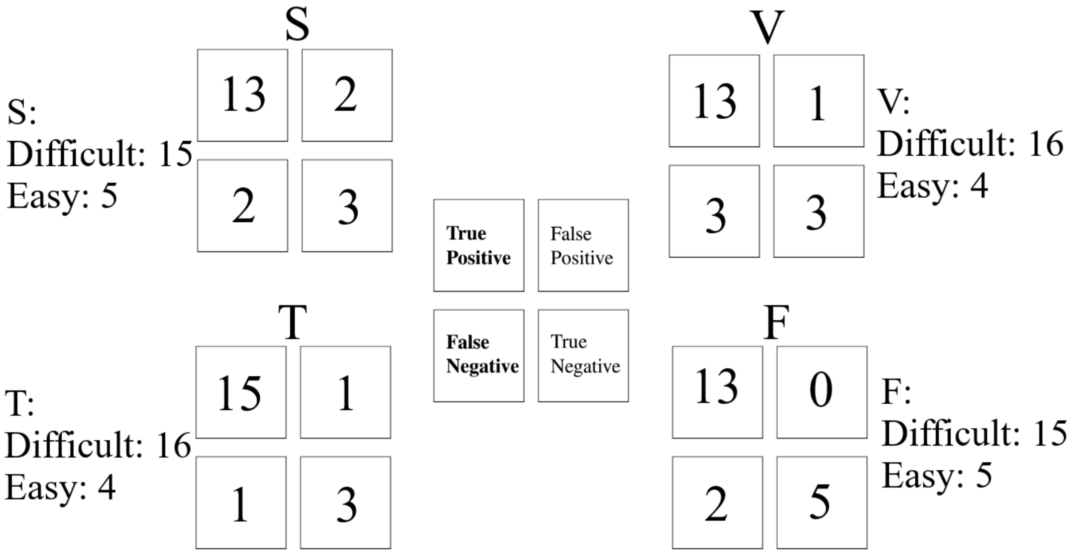

Figure 6. Right-hemisphere: Confusion matrices of our model during the storytelling experiment for speaker (S), video-chat (V), Telenoid (T), and in-person (F) media. In this figure, "Difficult" and "Easy" represent the number of participants whose self-assessed responses indicated "difficult" or "easy" story content. Results of the participants' self-assessment responses to difficulty of the story content identified that fifteen and five participants considered the story content difficult and easy in the speaker and the in-person. These numbers were sixteen and four in the video-chat and the Telenoid settings.

case of speaker (Figure 6 S, True Positive) and video-chat (Figure 6 S, True Positive). Whereas it misidentified one "easy" cases as "difficult" content in case of Telenoid (Figure 6 T, False Positive block) and video-chat (Figure 6 V, False Positive block), such a misestimation was two in case of the speaker (Figure 6 S, False Positive block). Last, its misclassification of "difficult" content as "easy" was highest in case of video-chat (Figure 6 V, False Negative block).

Last, whereas we observed significant correlations (Figure 7) between the participants' self-assessed responses to difficulty of the story content and their CLs in speaker (r = .47, $p < .05$, uncorrected), Telenoid (r = .47, $p < .05$, uncorrected), and face-to-face (r = .47, $p < .05$, uncorrected) settings, it was non-significant in the case of video-chat (r = .38, p = .10, uncorrected).

3 Self-Assessed Responses to Difficulty of the Story Content

Kruskal-Wallis (Figure 8) indicated non-significant defference between participants' self-assessed responses to difficulty of the story content in our media settings (p = .68, H(3, 79) = 1.53, r = .14, M_S = 5.45, SD_S = 1.67, M_V = 5.40, SD_V = 1.50, M_T = 5.55, SD_T = 1.36, M_F = 5.80, SD_F = 1.88).

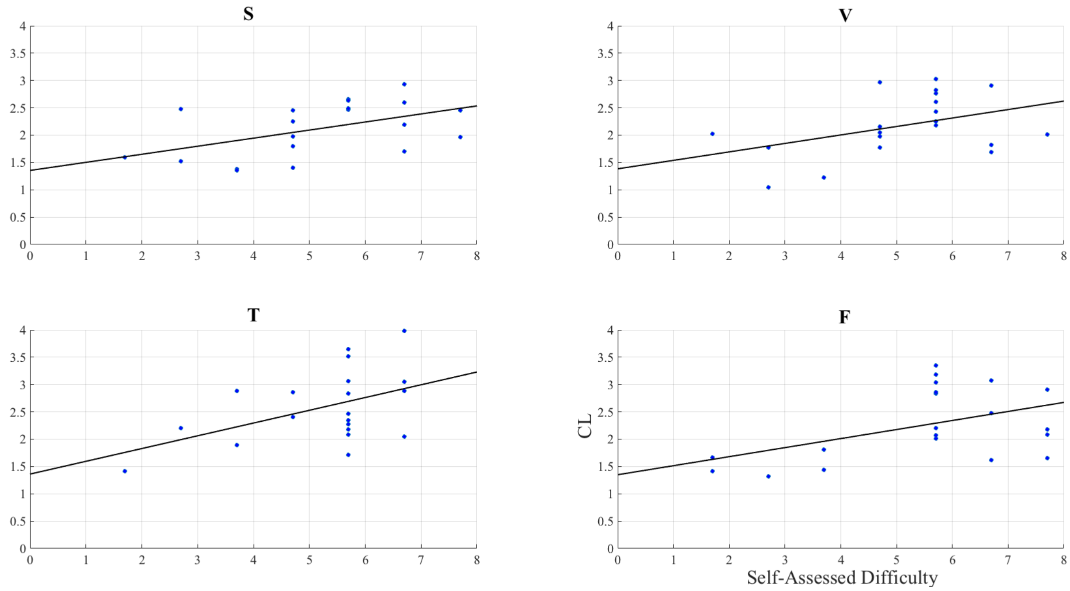

Figure 7. Right PFC: Spearman correlation between self-assessed responses of participant to difficulty of the story content and their CLs in speaker (S), video-chat (V), Telenoid (T), and face-to-face (F) settings.

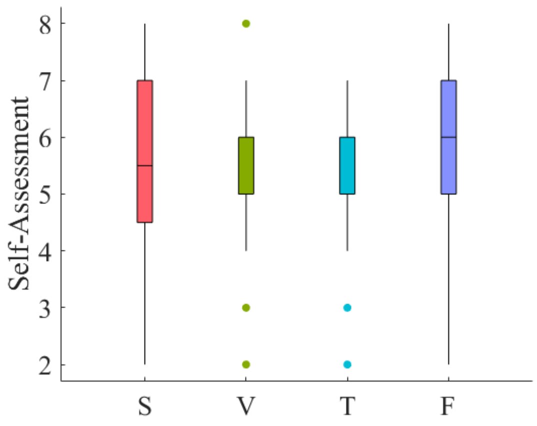

Figure 8. Kruskal-Wallis test on participants' self-assessed responses to their perceived difficulty of the story content was non-significant. $M_S = 5.45$, $SD_S = 1.67$, $M_V = 5.40$, $SD_V = 1.50$, $M_T = 5.55$, $SD_T = 1.36$, $M_F = 5.80$, $SD_F = 1.88$
